# Mesenchymal stem cells alleviate the early brain injury of subarachnoid hemorrhage partly by suppression of Notch1-dependent neuroinflammation: involvement of Botch

**DOI:** 10.1186/s12974-019-1396-5

**Published:** 2019-01-15

**Authors:** Wenchao Liu, Ran Li, Jian Yin, Shenquan Guo, Yunchang Chen, Haiyan Fan, Gancheng Li, Zhenjun Li, Xifeng Li, Xin Zhang, Xuying He, Chuanzhi Duan

**Affiliations:** Department of Neurosurgery, Zhujiang Hospital, Southern Medical University, The National Key Clinical Specialty, The Engineering Technology Research Center of Education Ministry of China, Guangdong Provincial Key Laboratory on Brain Function Repair and Regeneration, Guangzhou, 510282 China

**Keywords:** Subarachnoid hemorrhage, Mesenchymal stem cells, Early brain injury, Neuroinflammation, Notch1, Botch

## Abstract

**Background:**

Activated microglia-mediated neuroinflammation has been regarded as an underlying key player in the pathogenesis of subarachnoid hemorrhage (SAH)-induced early brain injury (EBI). The therapeutic potential of bone marrow mesenchymal stem cells (BMSCs) transplantation has been demonstrated in several brain injury models and is thought to involve modulation of the inflammatory response. The present study investigated the salutary effects of BMSCs on EBI after SAH and the potential mechanism mediated by Notch1 signaling pathway inhibition.

**Methods:**

The Sprague-Dawley rats SAH model was induced by endovascular perforation method. BMSCs (3 × 10^6^ cells) were transplanted intravenously into rats, and *N*-[*N*-(3,5-difluorophenacetyl-l-alanyl)]-S-phenylglycine *t*-butyl ester (DAPT), a Notch1 activation inhibitor, and Notch1 small interfering RNA (siRNA) were injected intracerebroventricularly. The effects of BMSCs on EBI were assayed by neurological score, brain water content (BWC), blood-brain barrier (BBB) permeability, magnetic resonance imaging, hematoxylin and eosin staining, and Fluoro-Jade C staining. Immunofluorescence and immunohistochemistry staining, Western blotting, and quantitative real-time polymerase chain reaction were used to analyze various proteins and transcript levels. Pro-inflammatory cytokines were measured by enzyme-linked immunosorbent assay.

**Results:**

BMSCs treatment mitigated the neurobehavioral dysfunction, BWC and BBB disruption associated with EBI after SAH, reduced ionized calcium binding adapter molecule 1 and cluster of differentiation 68 staining and interleukin (IL)-1 beta, IL-6 and tumor necrosis factor alpha expression in the left hemisphere but concurrently increased IL-10 expression. DAPT or Notch1 siRNA administration reduced Notch1 signaling pathway activation following SAH, ameliorated neurobehavioral impairments, and BBB disruption; increased BWC and neuronal degeneration; and inhibited activation of microglia and production of pro-inflammatory factors. The augmentation of Notch1 signal pathway agents and phosphorylation of nuclear factor-κB after SAH were suppressed by BMSCs but the levels of Botch were upregulated in the ipsilateral hemisphere. Botch knockdown in BMSCs abrogated the protective effects of BMSCs treatment on EBI and the suppressive effects of BMSCs on Notch1 expression.

**Conclusions:**

BMSCs treatment alleviated neurobehavioral impairments and the inflammatory response in EBI after SAH; these effects may be attributed to Botch upregulation in brain tissue, which subsequently inhibited the Notch1 signaling pathway.

**Electronic supplementary material:**

The online version of this article (10.1186/s12974-019-1396-5) contains supplementary material, which is available to authorized users.

## Background

Subarachnoid hemorrhage (SAH) represents a life-threatening cerebrovascular disease with high mortality and morbidity rates [1, 2]. Although considerable efforts have shed light on the mechanisms involved in pathological events associated with SAH, the potential pathophysiological mechanisms are still poorly understood. Angiographic vasospasm and delayed ischemic neurological deficit were once regarded as the major cause of the unfavorable prognosis after SAH [3]; however, clinical trials revealed that the poor prognosis associated with SAH was not improved after use of anti-vasospastic drugs [4, 5]. Recent advances in SAH research demonstrated that early brain injury (EBI), which refers to whole brain injury within the first 72 h post-SAH, was deemed as one of the major causes of mortality and unfavorable outcomes in SAH patients [6, 7]. As reported, multiple physiological abnormalities during EBI trigger the activation of inflammatory cascades, which result in blood-brain barrier (BBB) disruption and neuronal degeneration [8, 9]. Therefore, it is critical to explore the mechanisms of neuroinflammation and to discover effective therapies targeting the neuroinflammatory processes in SAH [10, 11].

Mesenchymal stem cells (MSCs) have widely been used in experimental and clinical studies and have been demonstrated to have therapeutic potential in various system diseases such as ischemic stroke [12, 13], preterm brain injury [14], multiple sclerosis [15, 16], cardiac disease [17], and traumatic brain injury [18]. Exogenously transplanted MSCs migrated to damaged tissue sites, where they differentiated into several different lost or injured cell types and modulate immune responses [19, 20]. However, increasingly, recent evidence suggests that the protective effects of MSCs are mediated in a paracrine manner [21, 22]. It has been reported that MSCs administration inhibited microglia activation in several central nervous system (CNS) diseases [23, 24]. Although the beneficial effects of bone marrow mesenchymal stem cells (BMSCs) transplantation at 14 days [25, 26] and at 21 days [27] post-SAH have previously been reported, to date and to the best of our knowledge, no studies have investigated the effects of BMSCs treatment on EBI after SAH or the possible underlying mechanisms.

The Notch1 signaling pathway plays an essential role in regulating cell fate decisions and in tissue homeostasis [28–30]. Upon binding to its ligands Delta-1 or Jagged-1, the Notch1 receptor is cleaved by disintegrin-metalloprotease tumor necrosis factor (TNF)-α-converting enzyme and γ-secretase complex, which releases the Notch1 intracellular receptor domain (NICD) from the membrane [31]. The NICD then translocates to the nucleus, where it interacts with the transcription factor recombining binding protein suppressor of hairless (RBP-Jκ), as wells as other regulators to modulate a variety of cellular metabolisms, such as cell proliferation and differentiation [32, 33]. In addition, increasing evidence indicates that Notch1 signaling may also play an essential role in activation of the inflammatory response in various diseases [34, 35]. Recent studies demonstrated that Notch1 signaling not only contributed to activated microglia-mediated neuroinflammation [36–38] but also aggravated neuronal degeneration [39], at least in part, via the nuclear factor-κB (NF-κB) signaling pathway during CNS diseases [40, 41]. MSCs transplantation has been demonstrated to suppress Notch1 signaling in ischemic stroke, inflammatory bowel disease, and lupus [42–44]. However, whether MSCs administration inhibits the Notch1 signaling pathway in EBI after SAH is unclear.

Cation transport regulator homolog 1 (Chac1) was identified as neuroprotective gene 7 (*NPG7*), recently renamed Botch-using functional cloning strategy [45]. As an endogenous blocker of Notch1 receptor, Botch inhibits the S1 furin-like cleavage of Notch1, thus promoting maintenance of full-length Notch1 [46]. Recent studies reported that Botch promoted neurogenesis through antagonizing the maturation of Notch1 during neocortical development and intracerebral hemorrhage [47, 48]. However, whether Botch participates in the suppressive effects of BMSCs on Notch1 expression remains unexplored. Thus, we hypothesized that BMSCs treatment may alleviate the inflammatory response and neurodegeneration via inhibiting the Notch1 signaling pathway in EBI after SAH. We also investigated whether BMSCs treatment inhibited Notch1 activation in EBI after SAH through a mechanism involving the upregulation of Botch in brain tissue.

## Methods

### Animals

Adult male Sprague-Dawley rats (280–320 g) were purchased from the Animal Experiment Center of Southern Medical University (Guangzhou, China). The animals were bred and housed in controlled temperature and humidity conditions (22 ± 1 °C; 40–60%) with a 12:12 dark/light cycle (lights on at 07:00 h; off at 21:00 h), and food and water were available ad libitum. All experimental procedures were approved by the Southern Medical University Ethics Committee and were performed in accordance with the guidelines of the National Institutes of Health on the care and use of animals.

### Experimental design

The experiments were performed as follows (Additional file 1).

#### Experiment 1

The impact of BMSCs administration on brain injury and neuroinflammation were assessed at 24 and 72 h after SAH. Neurological evaluation, brain water content (BWC), BBB permeability, magnetic resonance imaging (MRI), and hematoxylin and eosin (H&E) staining were conducted at 24 and 72 h post-SAH. Neuronal degeneration and activated microglia-mediated neuroinflammation were separately examined by Fluoro-Jade C (FJC) staining, immunofluorescence (IF) staining, and enzyme-linked immunosorbent assay (ELISA). A total of 167 rats were randomly assigned into 5 groups: Sham 24 h group (*n* = 24), SAH 24 h + phosphate buffer solution (PBS) group (*n* = 31), SAH 24 h + BMSCs group (*n* = 29), Sham 72 h group (*n* = 24), SAH 72 h + PBS group (*n* = 30), and SAH 72 h + BMSCs group (*n* = 29).

#### Experiment 2

To evaluate the time course of endogenous Notch1 and NICD expression after SAH, Western blotting was performed to detect Notch1 and NICD expression in the left hemisphere of each group. Double-labeling immunofluorescence of NICD and ionized calcium binding adapter molecule1 (Iba1)-positive microglia at 24 h post-SAH were conducted to determine the cellular localization of NICD. A total of 52 rats were randomly divided into following groups: Sham (*n* = 6), SAH 3 h (*n* = 6), SAH 6 h (*n* = 7), SAH 12 h (*n* = 8), SAH 24 h (*n* = 9), SAH 48 h (*n* = 8), and SAH 72 h (*n* = 8).

#### Experiment 3

To evaluate the effects of *N*-[*N*-(3,5-difluorophenacetyl-l-alanyl)]-S-phenylglycine *t*-butyl ester (DAPT), a γ-secretase inhibitor, on brain injury and neuroinflammation at 24 h post-SAH, Western blotting, IF staining, and immunohistochemistry (IHC) staining were used to assess the inhibitory effect of DAPT on Notch1 signaling pathway. Following injection of DAPT, neurological evaluation, BWC, FJC, and NeuN staining and ELISA were performed at 24 h post-SAH as follows: Sham group (*n* = 24), SAH 24 h group (*n* = 31), SAH 24 h + dimethylsulfoxide (DMSO) group (*n* = 31), and SAH 24 h + DAPT group (*n* = 29).

#### Experiment 4

To assess the impact of knockdown of endogenous Notch1 in vivo on brain injury and neuroinflammation after 24 h SAH, we assessed the inhibitory effect of Notch1 small interfering RNA (siRNA) on Notch1 signaling pathway by using quantitative real-time polymerase chain reaction (qRT-PCR), Western blotting, IF, and IHC staining. Notch1 siRNA administration was conducted at 48 h before modeling, and then neurological evaluation, BWC, FJC staining, NeuN staining, and ELISA were performed at 24 h post-SAH. A total of 130 rats were randomly divided into 4 groups: Sham (*n* = 27), SAH 24 h (*n* = 35), SAH 24 h + scramble siRNA (*n* = 35), and SAH 24 h + Notch1 siRNA (*n* = 33).

#### Experiment 5

To validate the hypothesis that the Notch1 signal pathway participates in the protective mechanism of BMSCs on EBI after SAH, we detected the expression of endogenous Notch1 using Western blotting, qRT-PCR, and IF staining at 24 and 72 h post-SAH. A total of 55 rats were randomly assigned into 5 groups: Sham (*n* = 9), SAH 24 h + PBS (*n* = 12), SAH 24 h + BMSCs (*n* = 11), SAH 72 h + PBS (*n* = 12), and SAH 72 h + BMSCs (*n* = 11).

#### Experiment 6

To detect the potential mechanism of the inhibitory effect of BMSCs on Notch1 signaling after SAH, Botch protein and mRNA levels were quantitatively analyzed at 24 and 72 h post-SAH with or without BMSCs treatment. Furthermore, we knocked down Botch expression in BMSCs and then transplanted them intravenously through the left femoral vein; efficiency of gene knockdown was assessed with Western blotting and qRT-PCR. The effects of BMSCs with attenuated Botch on neurobehavioral deficits and BWC were estimated at 24 h after SAH. A total of 56 rats were randomly divided into 3 groups: SAH + PBS (*n* = 19), SAH + BMSCs (*n* = 18), and SAH + BMSCs sh-Botch (*n* = 19).

### SAH rat model

The SAH model in rats was induced as described previously with small modifications [49]. In brief, with anesthetized rates by intraperitoneal injection of pentobarbital sodium (45 mg/kg) and maintained a core temperature at 37.5 °C. A 3–0 monofilament suture was advanced centripetally through the left external carotid artery (ECA), the common carotid artery, and the internal carotid artery (ICA) to perforate the intracranial bifurcation of the ICA. After SAH, the suture was withdrawn into the ECA then perfused the ICA. Sham-operated rats underwent the same procedures except that the suture was inserted beyond the point once resistance was felt. At the end of the surgical procedure, rats were allowed to recover on a heated blanket and then returned to separate cages with free access to food and water.

### BMSCs isolation, identification, and gene modification

BMSCs were isolated and cultured as previously described [50]. Briefly, the femora and tibias were dissected from both knees of adult male spontaneous hypertensive Sprague-Dawley rats weighing 80–100 g. The bone marrow plugs were then rinsed from the medullary cavity using a 10-mL syringe with Dulbecco’s modified Eagle’s medium-low glucose (Sigma-Aldrich, St. Louis, MO,USA) supplemented with 10% fetal bovine serum (Biocell, Rancho Dominguez, CA,USA). The bone marrow cell suspension was centrifuged at 400×*g* for 20 min to obtain mononuclear cells. The mononuclear cells were cultured in culture flasks with a density of 1 × 10^6^ cells/25 cm^2^ at 37 °C with 5% CO_2_. When the cells reached 90% confluency, the adherent cells were trypsinized and expanded. BMSCs passaged four times were used in the subsequent experiments. Typical positive BMSCs markers (CD29, CD44, and CD90) and a negative marker (CD45) were tested by flow cytometry analysis. Antibodies were purchased from BD Biosciences (555005, dilution 1:10; 550974, dilution 1:10; 551401, dilution 1:10; 551402, dilution 1:10 respectively, San Jose, CA, USA).

During the first passage, BMSCs were transfected with lentivirus at a multiplicity of infection of eight [51]. BMSCs transfected with Botch short hairpin RNA (shRNA) lentivirus were defined as BMSCs sh-Botch and transfected with mock lentivirus were defined as negative control (NC) group (BMSCs sh-NC). Polybrene (10 μg/mL) was incubated to achieve the optimal gene transfer, and qRT-PCR and Western blotting of Botch production confirmed the efficiency of gene transduction in BMSCs sh-Botch.

### BMSCs transplantation

BMSCs were delivered intravenously into rats within 1 h after induction of SAH as previously described. First, the femoral vein was dissected and exposed with blunt dissection. BMSCs (3 × 10^6^cells) were then suspended in 1 mL PBS and were injected slowly via the femoral vein for 5 min. The needle was then removed, the femoral vein was ligated, and the incision was closed carefully. As the control group, rats were infused with equal amount of PBS without BMSCs.

### Intracerebroventricular injection

Injection of DAPT and Notch1 siRNA into the lateral ventricle was performed as previously described [52]. Briefly, rats were anesthetized and placed on a stereotaxic frame. A midline skin incision was performed and a 10-μL microsyringe (Shanghai High Pigeon Industry & Trade Co., Ltd., Shanghai, China) was inserted into the left lateral ventricle via a burr hole, which was drilled according to the coordinates relative to bregma: 1.5 mm posterior, 1.0 mm lateral, and 3.2 mm below the horizontal plane of bregma. Following the manufacturer’s instructions, DAPT (Sigma-Aldrich) was dissolved in DMSO at 1 mg/mL. DAPT and vehicle (DMSO) were administered slowly at 1 μL/min into the left lateral ventricle after 90 min SAH [53]. The microsyringe was kept in place for an additional 5 min after administration and then withdrawn slowly. Lastly, the burr hole was closed by bone wax and the incision was sutured carefully. Notch1 siRNA (sc-270189, Santa Cruz Biotechnology, CA, USA) and scramble siRNA were separately dissolved in transfection reagent (Entranster TM-in vivo, 18668-11-1, Engreen Biosystem Co, Ltd., Beijing, China) at 0.45 μg/μL and then infused at 48 h before the induction of SAH.

### Neurological score evaluation and SAH grade

Neurological scores (neurobiological deficits) were evaluated by an independent investigator blinded to procedure information using the modified Garcia test [54, 55]. In the Garcia test, six sensorimotor tests including spontaneous activity, spontaneous movement of all limbs, forelimbs outstretching, climbing, touch of trunk, and vibrissae touch were assessed. Every test was scored as 0 to 3, and the total scores ranged from 0 to 18. Higher scores represent lighter neurological deficits.

Evaluation of SAH severity was performed blindly with an SAH grading system as previously reported. The basal surface of the brain was divided into six parts, and each part was scored 0 to 3 based on the amount of subarachnoid blood. Total scores ranged from 0 to 18 and mild SAH rats (total scores < 8) were subsequently eliminated from the study because there were no significant difference neurological impairments between sham and both treated and untreated mild SAH groups [55].

### MRI

At 24 and 72 h post-SAH, T_2_-weighted images were acquired using a 7.0-T small animal PharmaScan 70/16 MRI scanner (Bruker, MA, USA) according to the following parameters: repetition time (TR)/echo time (TE) = 4000/96 ms; scan time = 2 min; field of view 35 × 35 mm, matrix 256 × 256 mm; coronal slice thickness = 1.0 mm; and number of slices = 25 [56, 57].

### BWC

At 24 and 72 h post-SAH, brains were removed and divided quickly into four segments: left hemisphere, right hemisphere, cerebellum, and brain stem. Every segment was weighed immediately to obtain the wet weight (WW), and then dried in an oven for 72 h at 105 °C to obtain the dry weight (DW). The percentage of BWC = [(WW − DW)/WW] × 100%.

### BBB permeability

BBB disruption was evaluated on the basis of Evans Blue dye extravasation as previously described [58]. Briefly, 2% Evans Blue dye (4 mL/kg was injected intravenously after at 24 and 72 h post-SAH) was administered over 2 min into the left femoral vein and circulated for 2 h. Under deep anesthesia, the brains were harvested and separated immediately into the left and right cerebral hemispheres. Subsequently, the brain samples were weighed, homogenized in 50% trichloroacetic acid solution (2 mL), and centrifuged at 15,000×*g* for 30 min. The supernatant was mixed with ethanol (1:3) and incubated overnight at 4 °C. After centrifugation (15,000×*g* for 30 min), the resultant supernatant was spectrophotometrically quantified for the fluorescence intensity of Evans Blue dye at an excitation wavelength of 620 nm, an emission wavelength of 680 nm, and a band width of 10 nm.

### H&E staining

At 24 h and 72 h post-SAH, rats were anesthetized and transcardially perfused with 250 mL PBS (0.1 M, pH 7.4) followed by 500 mL 4% paraformaldehyde as previously described [59]. The brains were removed immediately and post-fixed in the same fixative for 24–48 h at 4 °C and embedded in paraffin and cut in serial coronal section (4 μm thickness). Sections were then deparaffinized and rehydrated, and were stained with hematoxylin for 5 min and eosin for 10 s. The slices were visualized and captured on a microscope (Leica-DM2500, Wetzlar, Germany).

### IF staining

Four-micron-thick brain coronal sections were prepared as described earlier, deparaffinized in xylene, rehydrated via alcohol gradients, and washed with PBS (0.01 M, pH 7.4). Following antigen retrieval, the sections were blocked in 5% bovine serum albumin for 30 min at room temperature (RT) and incubated with primary antibodies as follows: rabbit anti-activated Notch1 (1:200; Ab52301, Abcam, Cambridge, UK); goat anti-Iba1 (1:300; Ab5076, Abcam); mouse anti-CD68 (1:200; Ab31630, Abcam); rabbit anti-NeuN (1:200, 24307T, Cell Signaling Technology, Danvers MA, USA) overnight at 4 °C. The next day, the slices were washed with PBS and incubated with secondary antibodies: donkey anti-goat Alexa 488 (1:500; A11055, Invitrogen), donkey anti-rabbit Alexa 555 (1:500; A31572, Invitrogen), donkey anti-mouse Alexa 488 (1:500; A21202, Invitrogen), donkey anti-goat Alexa 555 (1:500; A21432, Invitrogen), goat anti-mouse Alexa 555 (1:500; A21422, Invitrogen), and donkey anti-rabbit Alexa 488 (1:500; A21206, Invitrogen) for 1 h at RT. After washing with PBS three times, the sections were stained by 4′6-diamidino-2-phenylindole for 10 min at room temperature and observed under a fluorescence microscope (Leica-DMI8, Leica Microsystems, Wetzlar, Germany).

### IHC staining

The deparaffinized and rehydrated coronal sections (4 μm thickness) were prepared as described earlier. Endogenous peroxidase activity was quenched using 3% H_2_O_2_ for 10 min at RT. Sections were then blocked in 5% bovine serum albumin for 20 min and were incubated overnight at 4 °C with the following primary antibodies: rabbit anti-RBP-Jκ (1:200; 5313T, Cell Signaling Technology) and rabbit anti-Hes-1 (1:200; 11988s, Cell Signaling Technology). Brain slices were then washed with PBS and incubated with biotinylated goat anti-rabbit IgG (1:100; ZSGB-Bio, Beijing, China) for 10 min at RT. Next, brain slices were incubated with horseradish peroxidase–streptavidin reagent for 10 min and developed using diaminbenzidine peroxidase substrate. Images were captured on a light microscope (Leica-DM2500, Wetzlar, Germany).

### FJC staining

FJC staining was performed to identify degenerated neurons as previously reported [59]. Briefly, brain sections were washed with alcohol solution (1% sodium hydroxide in 80% ethanol) followed by 70% ethanol and then immersed in 0.06% potassium permanganate solution for 10 min. Subsequently slides were transferred into a 0.0001% FJC (AG325, Millipore, Darmstadt, Germany) working solution for 30 min then cleared in xylene and coverslipped with permount TM mounting medium (Cwbiotech, Guangzhou, China). Images were obtained using a fluorescence microscope (Leica-DMI8, Leica Microsystems, Wetzlar, Germany).

### Western blotting

At 24 and 72 h post-SAH, rats were transcardially perfused with ice-cold PBS (0.1 M, pH 7.4) and the ipsilateral cortex was quickly dissected for western blot analysis as described previously [60]. Briefly, protein samples from the left cerebral cortex or BMSCs were isolated and prepared using RIPA lysis buffer (Cwbio, Guangzhou, China) and the total protein concentration was measured with a bicinchoninic acid protein assay kit (Genecopoeia, Rockville, MD, USA). Equal amounts of protein (50 μg) from different samples were separated by sodium dodecyl sulfate polyacrylamide gel electrophoresis (Cwbio, Guangzhou, China) and transferred to a polyvinylidene difluoride filter membrane. Membranes were then blocked in 5% non-fat milk dissolved in Tris-buffered saline with 0.1% Tween20 for 2 h and membranes were incubated with the following primary antibodies: mouse anti-Notch1 (1:1000, SC-376403, Santa Cruz Biotechnology); rabbit anti-activated Notch1 (1:1000, Ab52301, Abcam); mouse anti-RBP-Jκ (1:1000, SC-271128, Santa Cruz Biotechnology); rabbit anti-Hes-1 (1:1000, Ab108937, Abcam); rabbit anti-NF-κB P65 (1:1000, #3033, Cell Signaling Technology); rabbit anti-phospho-NF-κB P65 (1:1000, #8242, Cell Signaling Technology); mouse anti-Botch1 (1:500, N116/14, NeuroMeb, Davis, CA USA); and rabbit anti-β-actin (1:1000, #8475, Cell Signaling Technology) overnight at 4 °C and then washed for 10 min three times in Tris-buffered saline with 0.1% Tween20. Afterwards, the blots were incubated with secondary antibodies: peroxidase-conjugated goat anti-rabbit IgG and goat anti-mouse IgG (1:10000, Bioss, Beijing, China) and identified using the ECL Western blotting detection system (Millipore, Darmstadt, Germany). The intensities of blots were analyzed with ImageJ software (ImageJ 1.5, National Institutes of Health, Bethesda, MD, USA). β-actin was used as the internal loading control.

### qRT-PCR

Rat brain samples were homogenized in Trizol reagent (Sigma-Aldrich) according to the manufacturer’s instructions. Total RNA was isolated and reverse transcribed to cDNA with a primescript RT reagent kit (RR047A, Takara Bio Inc., Shiga, Japan). For qRT-PCR, the 7500 Real-Time PCR thermocycler (Applied Biosystem) was used. Real-time RT-PCR was performed in a total volume of 10 μL containing 1 μL of cDNA, 0.6 μL of primers, and 8.4 μL of SYBR Green PCR Master Mix (RR820A, Takara Bio Inc.). The program steps for amplification were pre-denaturation at 95 °C for 30 s, 40 cycles of 95 °C for 3 s, and extension at 60 °C for 30 s. The mean levels of target genes were calculated using the relative normalized expression with β-actin used as internal control. The primer sequences are listed as Table 1.

**Table 1:** Real-time PCR primers used for quantification of mRNA expression in this study

Primer name	Sequence (5′ → 3′)	
Notch1	Forward	GTGTGTGAAAAGCCCGTGTC
	Reverse	GCACAAGGTTCTGGCAGTTG
RBP-Jκ	Forward	CACGTTCCATGAGGGAGTGG
	Reverse	TGGCATTTGTGTAATTTCCTTTGC
Hes-1	Forward	GCTGCTACCCCAGCCAGTG
	Reverse	GCCTCTTCTCCATGATAGGCTTTG
Botch	Forward	TGGATTTTCGGGTACGGCTC
	Reverse	GTCTGCCAAACGCAACAAGT
β-actin	Forward	TCAGCAAGCAGGAGTACGATG
	Reverse	GTGTAAAACGCAGCTCAGTAACA

### ELISA

At 24 h and 72 h post-SAH, rats were anesthetized and transcardially perfused with 250 mL PBS, the left hemisphere was harvested immediately and homogenized in 0.9% normal saline at 200 mg/mL and centrifuged at 12,000×*g* for 20 min. The supernatant was collected and stored in − 80 °C until further used. The concentrations of TNF-α, interleukin (IL)-6, IL-1β, and IL-10 in brain tissue lysates were analyzed using commercial ELISA kits (BMS625, BMS630, and BMS629, Invitrogen) according to the manufacturer’s instructions. The final concentration of cytokines was interpolated from the determined standard curve of absorbance.

### Statistical analysis

All data were shown as means ± standard error of the mean (SEM) and analyzed using SPSS version 19.0 software (SPSS, IBM, Armonk, NY, USA). After checking for normal distribution, the statistical significance of two groups was determined using Student’s *t* test or Mann-Whitney *U* test; one-way analysis of variance (ANOVA) followed by Tukey’s post-hoc test or Kruskal-Wallis followed by Dunn’s test were applied for comparison of multiple groups; *P* < 0.05 was considered statistically significant. Cartograms were drawn using GraphPad Prism 5 software (GraphPad Software, Inc., San Diego, CA, USA).

## Results

### Characterization of rat BMSCs and representative macrographs

**Fig. 1:**
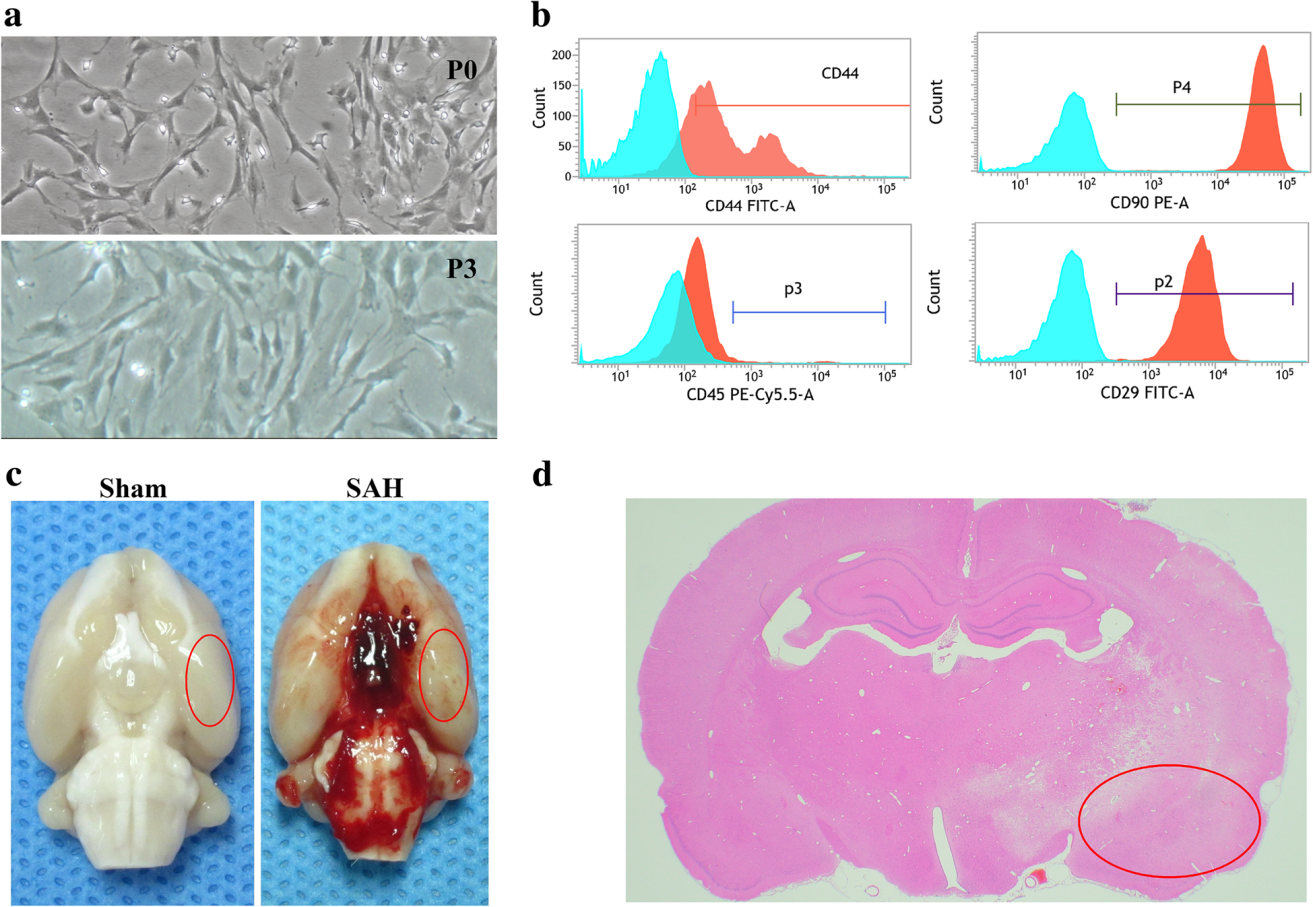
Identification of rat BMSCs and representative image of subarachnoid hemorrhage (SAH) model. Representative micrographs of BMSCs cultured at passage 0 (P0) and passage 3 (P3) (**a**). Flow cytometry analysis indicating positive BMSCs markers (CD29, CD44, CD90) and a negative marker (CD45) (**b**). Representative picture of brains from the Sham and SAH groups (**c**). A schematic indicating the optimal brain region for immunochemistry staining, qRT-PCR and western blotting (red circle) (**d**). CD, cluster of differentiation

Flow cytometry was used to characterize BMSCs by determining surface antigens expression (Fig. 1a, b). BMSCs were positive for CD90 (99.7%), CD29, (99.21%), and CD44 (74.27%), but were negative for CD45 (2.52%). Representative images of brains from rats in Sham and SAH groups are shown in (Fig. 1c). We specifically focused on the basal cortex of the left hemisphere (Fig. 1d).

### BMSCs transplantation attenuated neurobehavioral deficits and reduced histological damage at 24 and 72 h after SAH

**Fig. 2:**
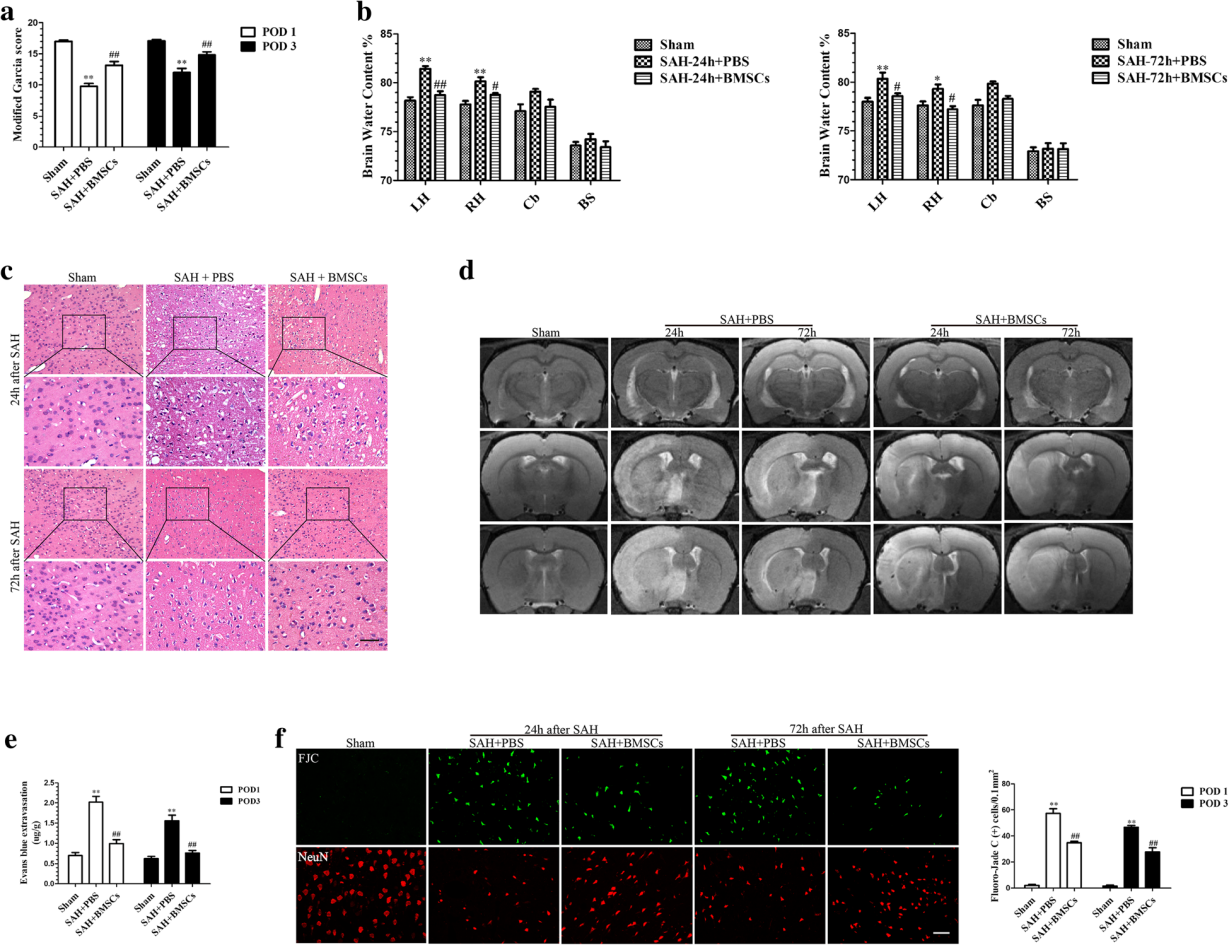
The effect of BMSCs transplantation on neurological function, brain edema, BBB disruption and neuronal degeneration after SAH. BMSCs treatment significantly improved neurological function (**a**) (*n* = 12/group) and reduced BWC both at 24 and 72 h post-SAH (**b**) (*n* = 6/group). Representative images of H&E staining (**c**) and T2-weighted MRI images (**d**) showing alterations in lesion volume after BMSCs treatment. The quantification of Evans Blue dye extravasation (**e**) (*n* = 6/group). Typical Fluoro-Jade C (FJC) and NeuN (neuronal marker) staining images and quantitative analysis of FJC-positive cells from the injured hemisphere (**f**) (*n* = 6/group). Data are expressed as the mean ± SEM. *P < 0.05, **P < 0.01 versus Sham, ^#^*P* < 0.05, ^##^*P* < 0.01 versus SAH + PBS group. Scale bar = 50 μm. *BS* brain stem, *Cb* cerebellum, *LH* left hemisphere, *RH* right hemisphere, *POD* post-operative day

Rats in the SAH + PBS groups showed severe behavioral deficits (Fig. 2a) and higher levels of cerebral edema (Fig. 2b), compared with rats in the Sham group at 24 h and at 72 h post-SAH. Transplantation of BMSCs improved behavioral performance and reduced edema in brain tissue, compared with SAH + PBS group. H&E staining (Fig. 2c) and MRI T_2_-weighted images (Fig. 2d) were also used to evaluate the effect of BMSCs administration at 24 h and 72 h post-SAH; the results indicated that BMSCs transplantations alleviated histological impairments.

### BMSCs administration alleviated disruption to the BBB and attenuated neuronal injury at 24 h and 72 h post-SAH

Evans Blue dye extravasation was significantly decreased in the left hemisphere following BMSCs treatment compared with the SAH + PBS group (Fig. 2e). To further investigate the protective effects of BMSCs treatment on EBI after SAH, we estimated neuronal loss and degeneration (Fig. 2f). At 24 h and 72 h post-SAH, FJC-positive neurons were frequently present in the cortical region of the ipsilateral hemisphere. Administration of BMSCs significantly decreased the number of FJC-positive neurons and increased the NeuN-positive neurons.

### Treatment with BMSCs inhibited microglia activation and decreased expression of inflammatory cytokines at 24 h and 72 h post-SAH

**Fig. 3:**
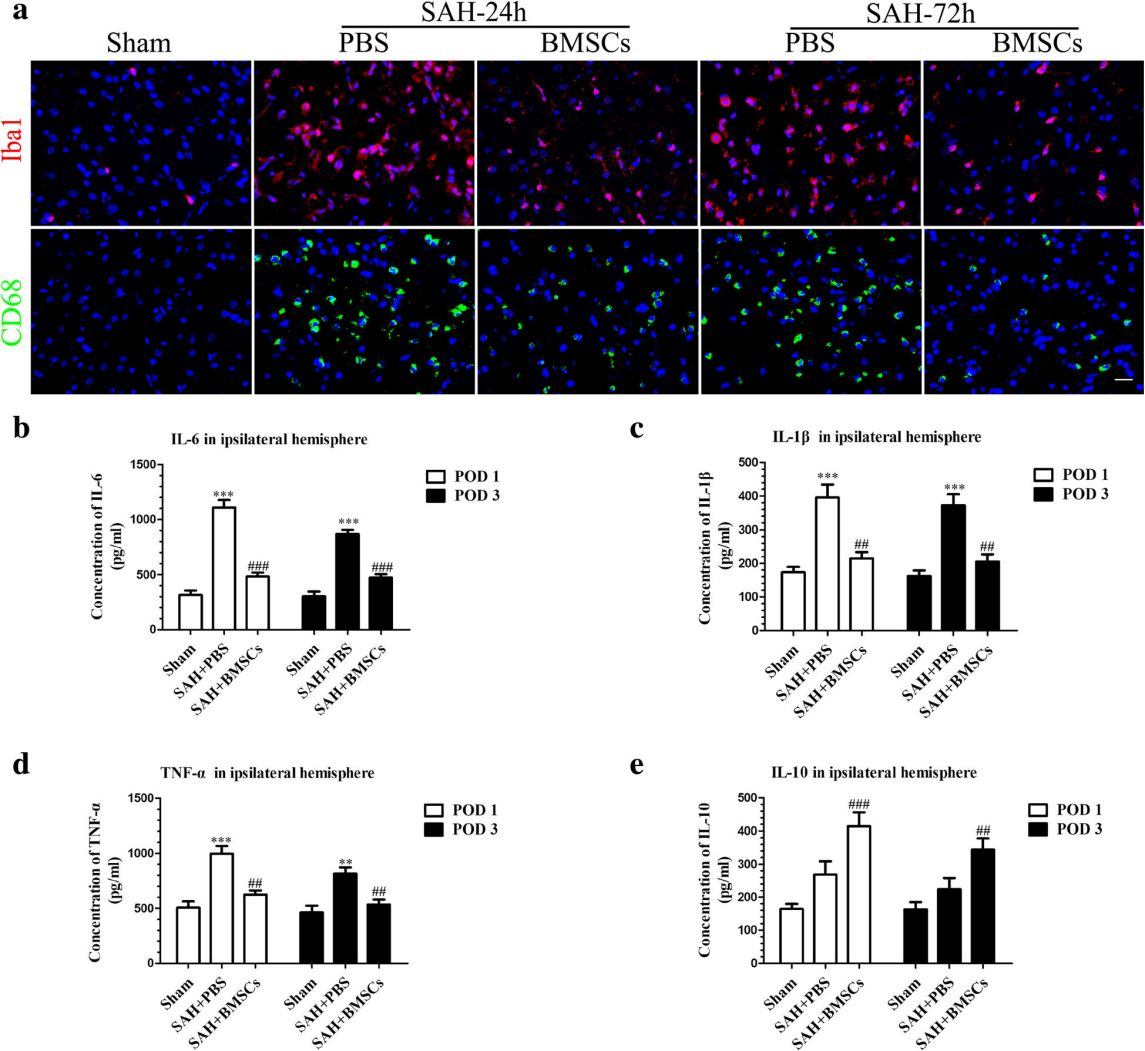
BMSCs treatment suppressed microglial activation and downregulated production of inflammatory cytokines IL-6, IL-1β, and TNF-α at 24 and 72 h post-SAH. Representative images of ionized calcium binding adapter molecule1 (Iba1)- and CD68-positive cells in the Sham, SAH + PBS, and SAH + BMSCs groups (**a**). BMSCs treatment decreased the expression of pro-inflammatory cytokines, IL-6 (**b**), IL-1β (**c**), and TNF-α (**d**) but elevated the expression of anti-inflammatory cytokine, IL-10 (**e**) in brain tissue both at 24 and 72 h post-SAH; *n* = 6 in each group; data are expressed as the mean ± SEM. **P < 0.01, ***P < 0.001 versus Sham, ^##^*P* < 0.01,^###^*P* < 0.001 versus SAH + PBS group. Scale bar = 50 μm

We performed co-staining of NeuN and CD68 (activated microglia/macrophage markers) as well as NeuN and Iba1 (labeled total microglia) to evaluate the microglia response after induction of SAH. Iba1- and CD68-positive cells were increased and NueN-positive cells were decreased after SAH (Additional file 2). BMSCs treatment decreased the Iba1-positive cells as well as CD68-positive cells in left hemisphere at 24 h and 72 h (Fig. 3a).

To further ascertain the effects of BMSCs on suppression of neuroinflammation, we examined several inflammatory cytokine levels in the ipsilateral hemisphere at 24 and 72 h post-SAH. BMSCs treatment significantly reduced the expression of the proinflammatory cytokines, IL-6, IL-1β, and TNF-α (Fig. 3b–d) but elevated the expression of the ant-inflammatory cytokine, IL-10 (Fig. 3e).

### Endogenous expression of Notch1 and its intracellular domain NICD after SAH

**Fig. 4:**
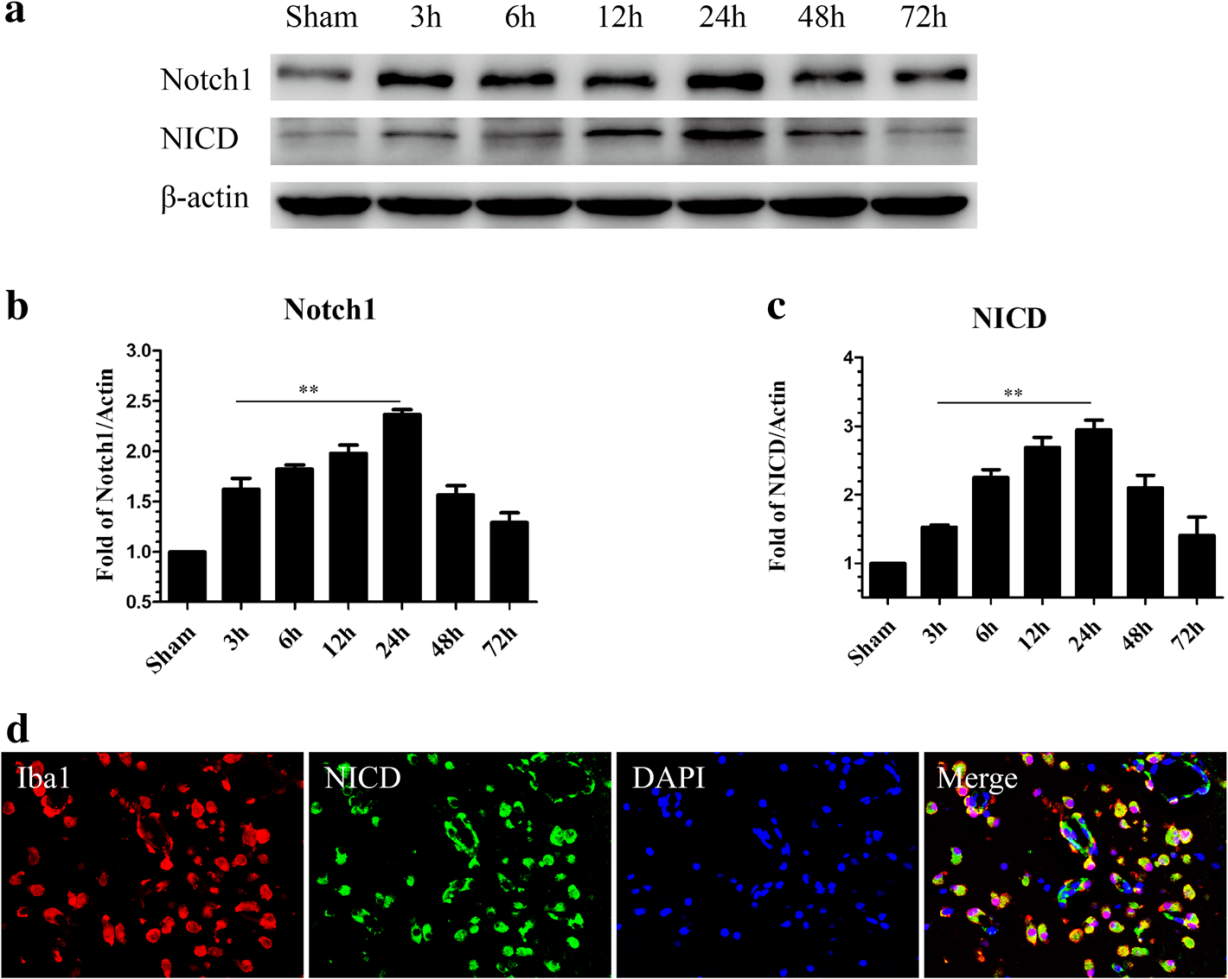
Time course of Notch1 and NICD expression in the left cortex after SAH. Representative images of Western blot data showing that endogenous Notch1 and NICD began to increase at 3 h and reached their highest expression at 24 h post-SAH (**a**). Quantification of Notch1 (**b**) and NICD (**c**) expression in the left cerebral cortex after SAH; *n* = 6 in each group; data are expressed as the mean ± SEM. **P < 0.01 versus Sham. Representative images of double immunofluorescence staining for NICD and Iba1 showing the co-localization of NICD with Iba1-positive microglia (**d**) in the left cerebral cortex at 24 h post-SAH. Scale bar = 50 μm. *NICD* Notch1 intracellular receptor domain

Western blotting was performed to investigate the endogenous expression of Notch1 and NICD in the left hemisphere with (3, 6, 12, 24, 48, and 72 h) and without SAH. Notch1 levels were elevated immediately after SAH, reached a peak at 24 h, and gradually declined at 48 h and 72 h (Fig. 4a, b). NICD levels increased from 12 h post-SAH, peaked at 24 h, and was reduced significantly at 72 h (Fig. 4a, c). Double immunofluorescence staining showed that NICD was predominantly expressed in microglia in the cortex of ipsilateral hemisphere at 24 h post-SAH (Fig. 4d).

### DAPT inhibited the Notch1 pathway at 24 h post-SAH

**Fig. 5:**
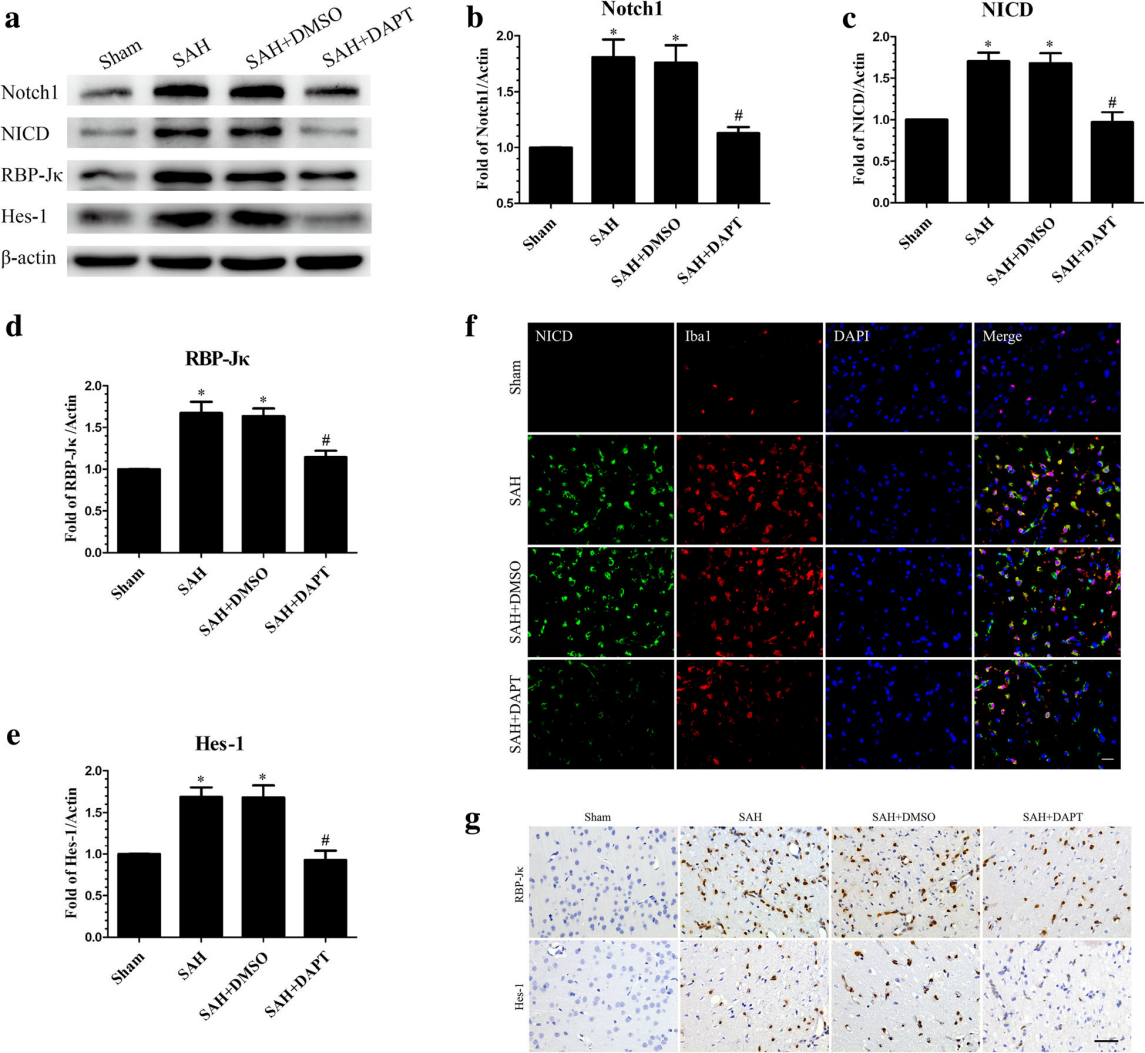
*N*-[*N*-(3,5-difluorophenacetyl-l-alanyl)]-S-phenylglycine *t*-butyl ester (DAPT) inhibited the protein expression of molecules in Notch1 pathway. Western blotting showing DAPT significantly reduced the expression of Notch1, NICD, RBP-Jκ, and Hes-1 (**a**), with quantitative analysis of Notch1 (**b**), NICD (**c**), RBP-Jκ (**d**), and Hes-1 (**e**) levels in left hemisphere at 24 h post-SAH; *n* = 6 in each group; data are expressed as the mean ± SEM. *P < 0.05 versus Sham, ^#^*P* < 0.05 versus SAH + DMSO group. Representative microphotographs of immunofluorescence staining for NICD and Iba1 (**f**), and immunohistochemistry staining for RBP-Jκ and Hes-1 (**g**) in the left cerebral cortex at 24 h post-SAH. Scale bar = 50 μm. RBP-Jκ: Recombining binding protein suppressor of hairless; Hes-1: Hes family basic helix loop helix transcription factor 1

Western blotting analysis showed that DAPT inhibited Notch1 expression at 24 h post-SAH. As NICD production is dependent on the enzymatic activity of the γ-secretase complex, DAPT administration significantly suppressed NICD accumulation. In the DAPT-treated group, RBP-Jκ and Hes family basic helix loop helix transcription factor 1 (Hes-1) were inhibited compared with the SAH + DMSO group (Fig. 5a–e). Consistent with the results of Western blotting, double immunofluorescence staining revealed that NICD expression in activated microglia was reduced following DAPT injection at 24 h post-SAH (Fig. 5f). IHC staining revealed that RBP-Jκ and Hes-1 were markedly increased at 24 h post-SAH, but these increases were negated after DAPT administration (Fig. 5g).

### Administration of DAPT reduced NF-κB phosphorylation, the microglia activation, and inflammatory factor production at 24 h post-SAH

**Fig. 6:**
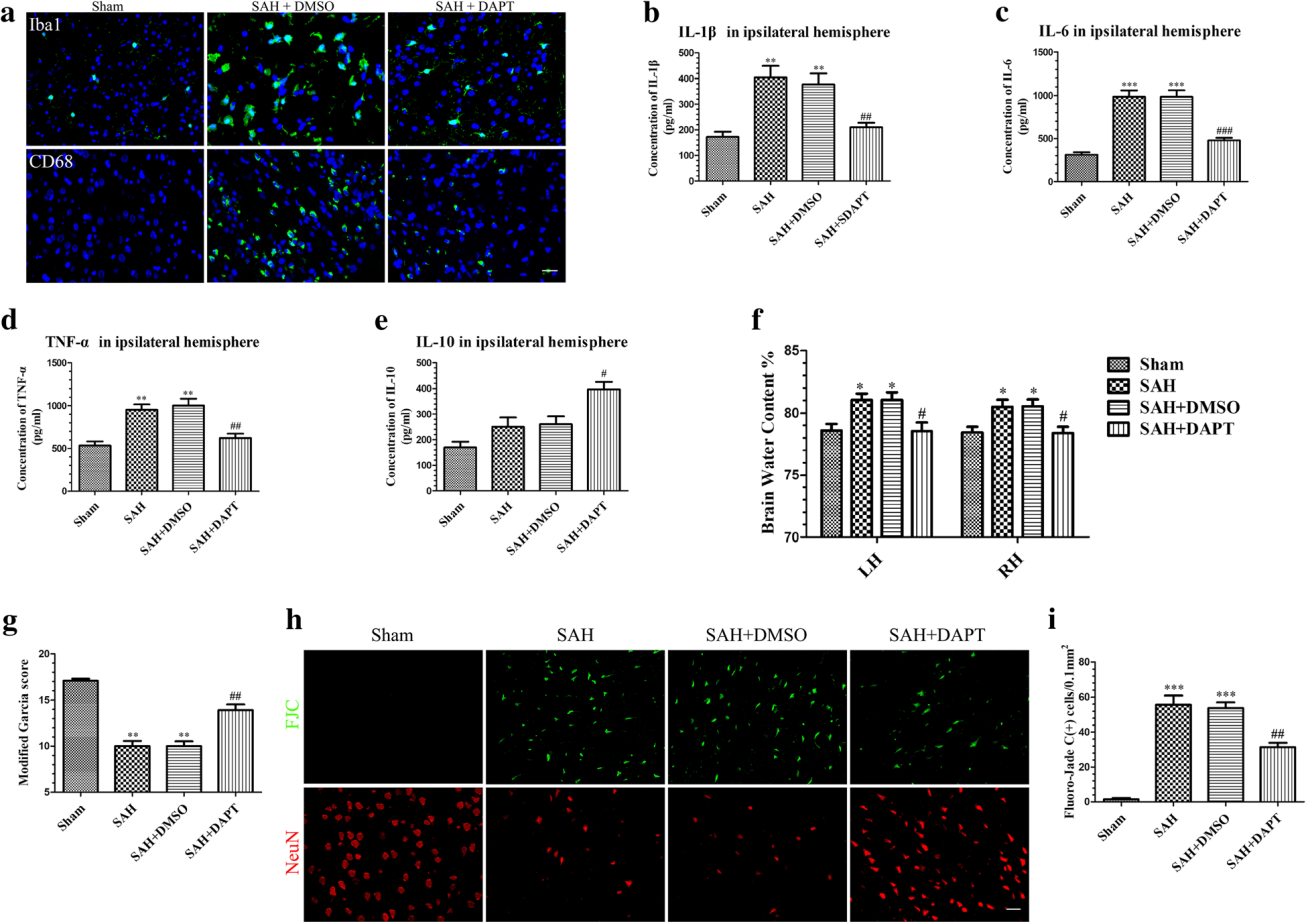
Effects of DAPT on microglial activation, inflammatory factors production, brain edema, neurobehavioral deficits, and neuronal degeneration in SAH rats. Representative images of Iba1- and CD68-positive microglia in the Sham, SAH + DMSO, and SAH + DAPT groups (**a**). ELISA results indicating the levels of the pro-inflammatory factors IL-1β (**b**), IL-6 (**c**), and TNF-α (**d**) and the anti-inflammatory factor IL-10 (**e**) in the ipsilateral cerebral tissue at 24 h post-SAH; *n* = 6 in each group. DAPT treatment markedly reduced BWC (**f**) (*n* = 6/group) in the left hemisphere and increased modified Garcia score (**g**) (*n* = 12/group). Representative FJC and NeuN staining images (**h**) and quantitative analysis of FJC-positive cells (**i**) (*n* = 6/group) in the left cortex after SAH with DAPT or DMSO treatment. Data are expressed as the mean ± SEM. *P < 0.05, **P < 0.01, ***P < 0.001 versus Sham, ^#^*P* < 0.05, ^##^*P* < 0.01, ^###^*P* < 0.001 versus SAH + DMSO group. Scale bar = 50 μm

Notch1 activation enhances the microglia-mediated neuroinflammation following SAH through NF-κB phosphorylation. Western blotting analysis showed that NF-κB phosphorylation was noticeably increased after SAH and that DAPT administration significantly attenuated NF-κB phosphorylation (Additional file 3A and B). Consistent with the foregoing result, IF staining revealed activation of microglia (Fig. 6a) were markedly inhibited after DAPT administration. In addition, DAPT treatment decreased expression of inflammatory cytokines, IL-1β, IL-6, and TNF-α (Fig. 6b–d), but upregulated the anti-inflammatory cytokine, IL-10 (Fig. 6e), at 24 h post-SAH.

### DAPT mitigates brain edema, neurodegeneration, and neurobehavioral deficits at 24 h post-SAH

To determine the protective function of DAPT at 24 h post-SAH, BWC, neurobehavioral deficits, and neurodegeneration were assessed. The results showed that DAPT significantly reduced BWC (Fig. 6f), improved neurobehavioral deficits (Fig. 6g), decreased the number of FJC-positive cells, and increased the number of NeuN-positive cells (Fig. 6h, i).

### Knockdown of Notch1 expression by RNA interference resulted in a reduction of Notch1 pathway-related molecules at 24 h post-SAH

**Fig. 7:**
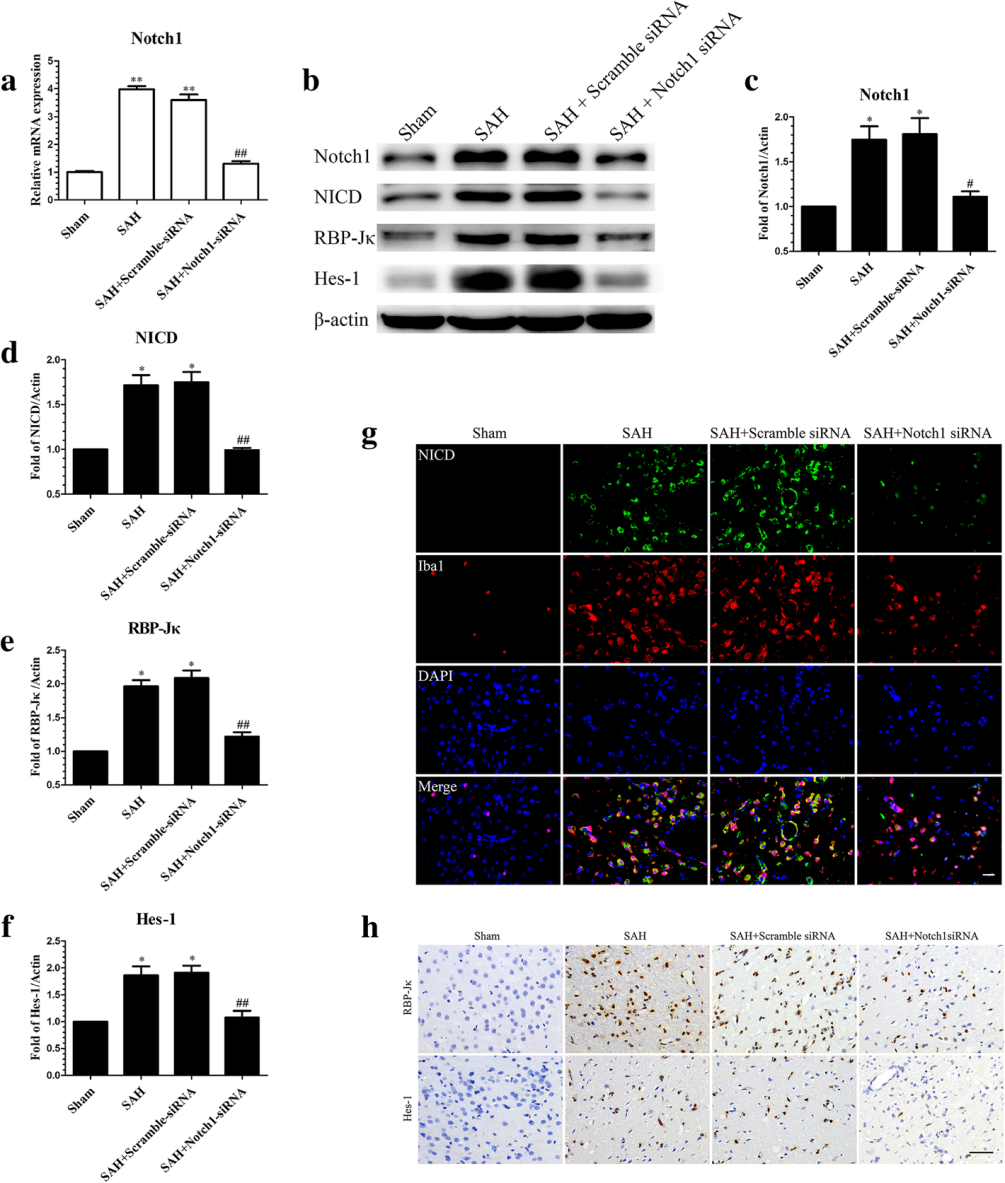
Effects of Notch1 siRNA on the expression of molecules in the Notch1 pathway at 24 h post-SAH. The qRT-PCR analysis of the efficiency of siRNA-mediated knockdown of Notch1 in ipsilateral cerebral cortex (**a**); *n* = 3 in each group; data are expressed as the mean ± SEM **P < 0.01 versus Sham, ^##^*P* < 0.01 versus SAH + Scramble siRNA group. The protein levels of Notch1 pathway-related agents were assessed by Western blot (**b**) and quantification of Notch1 (**c**), NICD (**d**), RBP-Jκ (**e**), and Hes-1 (**f**) at 24 h post-SAH; *n* = 6 in each group; data are expressed as the mean ± SEM. *P < 0.05 versus Sham, ^#^*P* < 0.05, ^##^*P* < 0.01 versus SAH + Scramble siRNA group. Representative images of NICD- and Iba1-positive cells (**g**) and immunohistochemistry staining for RBP-Jκ and Hes-1 (**h**) showing that Notch1 siRNA reduced the levels of NICD, RBP-Jκ and Hes-1 in ipsilateral cerebral cortex. Scale bar = 50 μm

To confirm the functional implications of Notch1 activation after SAH, Notch1 siRNA was used to knockdown Notch1 expression in brain tissue. The efficiency of siRNA-mediated knockdown of Notch1 was confirmed by qRT-PCR (Fig. 7a).

Western blotting was performed to estimate the protein levels of Notch1 and its related pathway molecules, as shown in (Fig. 7b–f). Knockdown of Notch1 reduced Notch1, NICD, RBP-Jκ, and Hes-1 compared with the SAH + Scramble siRNA and SAH group. Furthermore, IF staining for NICD (Fig. 7g) and IHC staining for RBP-Jκ and Hes-1 (Fig. 7h) showed that NICD, RBP-Jκ, and Hes-1 were inhibited following knockdown of Notch1.

### Notch1 knockdown inhibited NF-κB phosphorylation and reduced microglial activation and inflammatory cytokine infiltration at 24 h post-SAH

Phosphorylation of NF-κB was significantly increased at 24 h post-SAH, but Notch1 knockdown reduced this effect compared with SAH and SAH + Scramble siRNA groups (Additional file 3C and D).

**Fig. 8:**
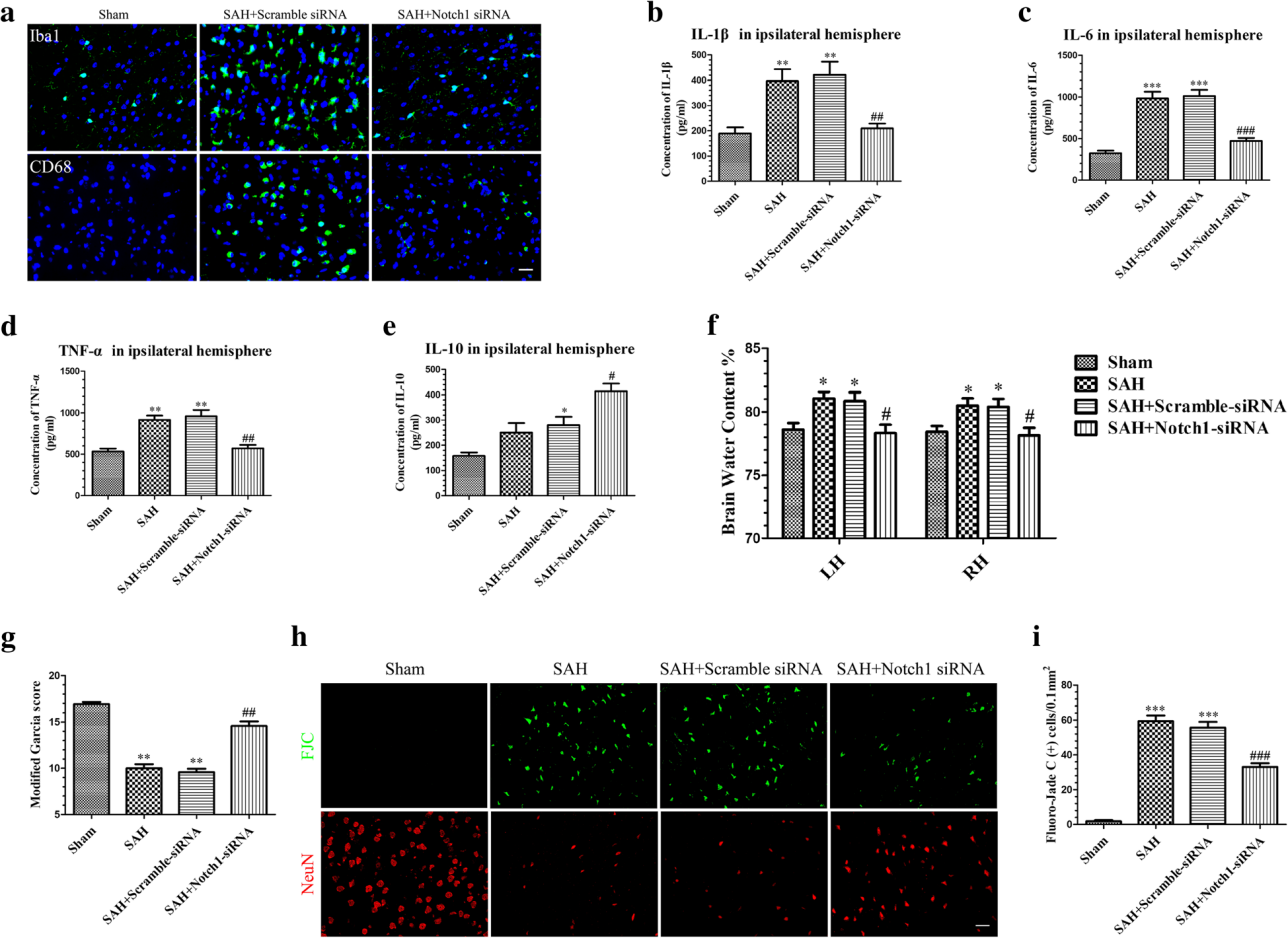
Effects of Notch1 siRNA on the microglial activation, inflammatory cytokines production, BWC, modified Garcia scores, and neuronal injury after 24 h SAH. Notch1 siRNA decreased Iba1- and CD68-positive cells in the left cerebral cortex at 24 h post-SAH (**a**). The protein levels of the proinflammatory cytokines IL-1β (**b**), IL-6 (**c**), and TNF-α (**d**) were decreased while the levels of the anti-inflammatory cytokine IL-10 (**e**) were increased in the SAH + Notch1 siRNA group compared with the SAH + Scramble siRNA group; *n* = 6 in each group. Notch1 siRNA treatment significantly alleviated BWC (**f**) (*n* = 6/group) in the left hemisphere and increased modified Garcia score (**g**) (*n* = 12/group) at 24 h post-SAH, representative FJC and NeuN staining images (**h**), and quantitative analysis of FJC-positive cells (**i**) (*n* = 6/group) in the left cortex after SAH with Notch1 siRNA injection. Data are expressed as the mean ± SEM. *P < 0.05, **P < 0.01, ***P < 0.001 versus Sham, ^#^*P* < 0.05, ^##^*P* < 0.01, ^###^*P* < 0.001 versus SAH + Scramble siRNA group. Scale bar = 50 μm

We next examined the effect of Notch1 knockdown on activated microglia. As shown in (Fig. 8a), Notch1 knockdown markedly suppressed Iba1- and CD68-positive cells at 24 h post-SAH. SAH-induced upregulation of pro-inflammatory cytokines IL-1β, IL-6, and TNF-α were also reduced (Fig. 8b–d), while the anti-inflammatory factor IL-10 was increased (Fig. 8e) following Notch1 knockdown.

### Notch1 knockdown ameliorated neurodegeneration and improved neurobiological deficits and brain edema

Notch1 knockdown markedly decreased BWC in cerebral tissue (Fig. 8f) and alleviated neurobiological deficits (Fig. 8g) compared with SAH and SAH + Scramble siRNA groups at 24 h post-SAH. The numbers of FJC-positive neurons were decreased (Fig. 8h, i) in the ipsilateral hemisphere after Notch1 siRNA injection, but the NeuN-positive neurons were increased.

### BMSCs treatment suppressed Notch1 signaling pathway activation at 24 h and 72 h post-SAH

**Fig. 9:**
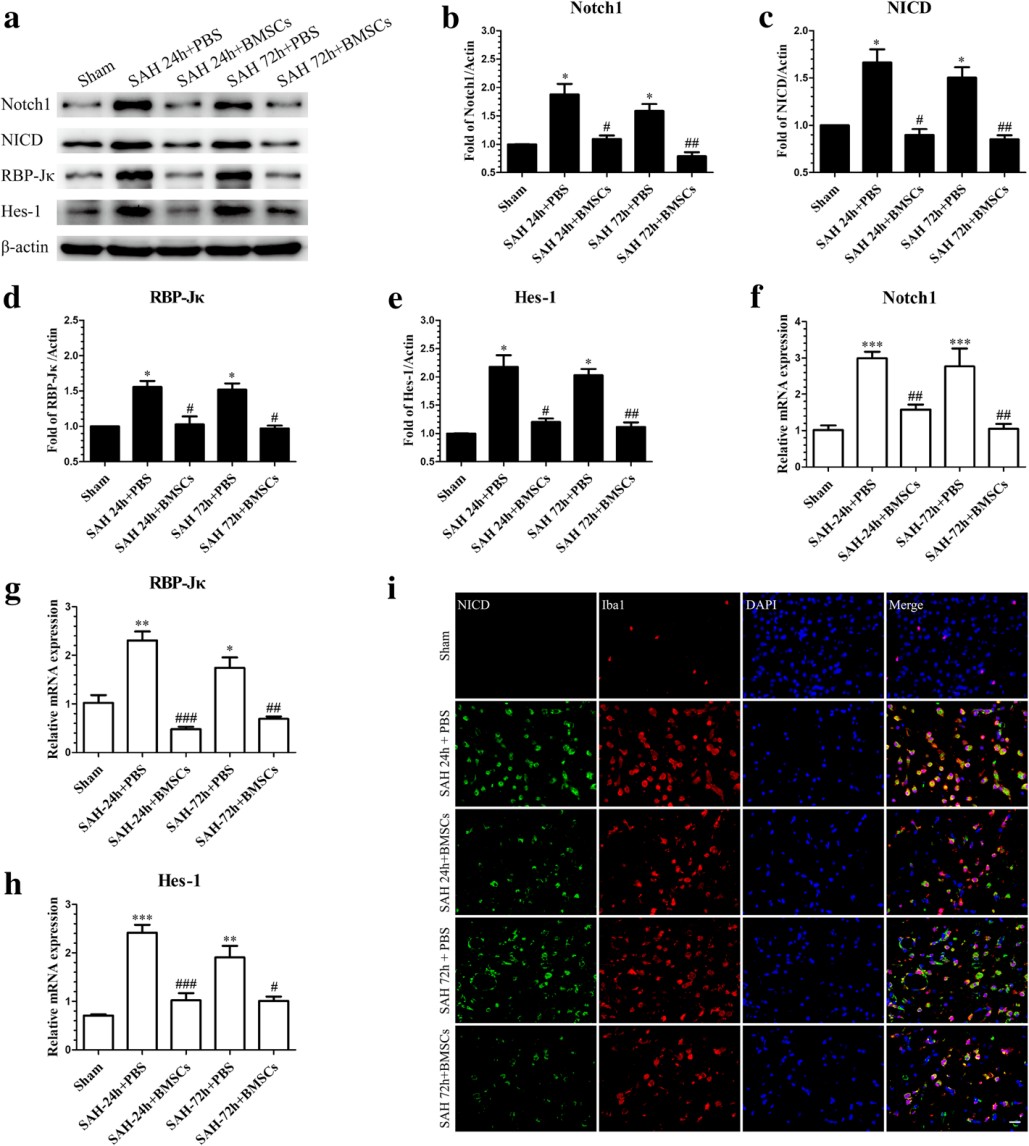
BMSCs transplantation inhibited SAH-induced upregulation of the Notch1 pathway-related molecules, Notch1, NICD, RBP-Jκ, and Hes-1 at 24 and 72 h post-SAH. Representative image of Western blotting (**a**) and quantification of Notch1 (**b**), NICD (**c**), RBP-Jκ (**d**), and Hes-1 (**e**) protein levels after BMSCs treatment at 24 and 72 h post-SAH; *n* = 6 in each group; data are expressed as the mean ± SEM. *P < 0.05 versus Sham, ^#^*P* < 0.05, ^##^*P* < 0.01 versus SAH+ PBS group. The qRT-PCR analysis of Notch1 (**f**), RBP-Jκ (**g**), and Hes-1 (**h**) mRNA levels at 24 and 72 h post-SAH; *n* = 3 in each group; data are expressed as the mean ± SEM. *P < 0.05, **P < 0.01, ***P < 0.001 versus Sham, ^#^*P* < 0.05, ^##^*P* < 0.01, ^###^*P* < 0.001 versus SAH + PBS group. Typical images of double-immunofluorescence staining for NICD and Iba1 (**i**) showing that BMSCs treatment suppressed NICD expression in Iba1-positive microglia at 24 and 72 h post-SAH. Scale bar = 50 μm

To explore the mechanisms involved in the protective effects of BMSCs on EBI after SAH, the expression of Notch1 signaling pathway-associated factors was determined after BMSCs treatment at 24 and 72 h post-SAH. Western blotting results indicated that BMSCs treatment significantly inhibited the production of Notch1, NICD, RBP-Jκ, and Hes-1 (Fig. 9a–e) compared with the SAH + PBS group. Consistent with the foregoing results, the mRNA levels of Notch1, RBP-Jκ, and Hes-1 were noticeably decreased after BMSCs treatment (Fig. 9f–h). IF staining showed that NICD immunofluorescence in activated microglia was reduced after BMSCs treatment (Fig. 9i). Finally, we also demonstrated that NF-κB phosphorylation after SAH was significantly inhibited following BMSCs treatment (Additional file 3E and F).

### Botch, an endogenous inhibitor of Notch1, was significantly increased after BMSCs treatment at 24 h post-SAH

**Fig. 10:**
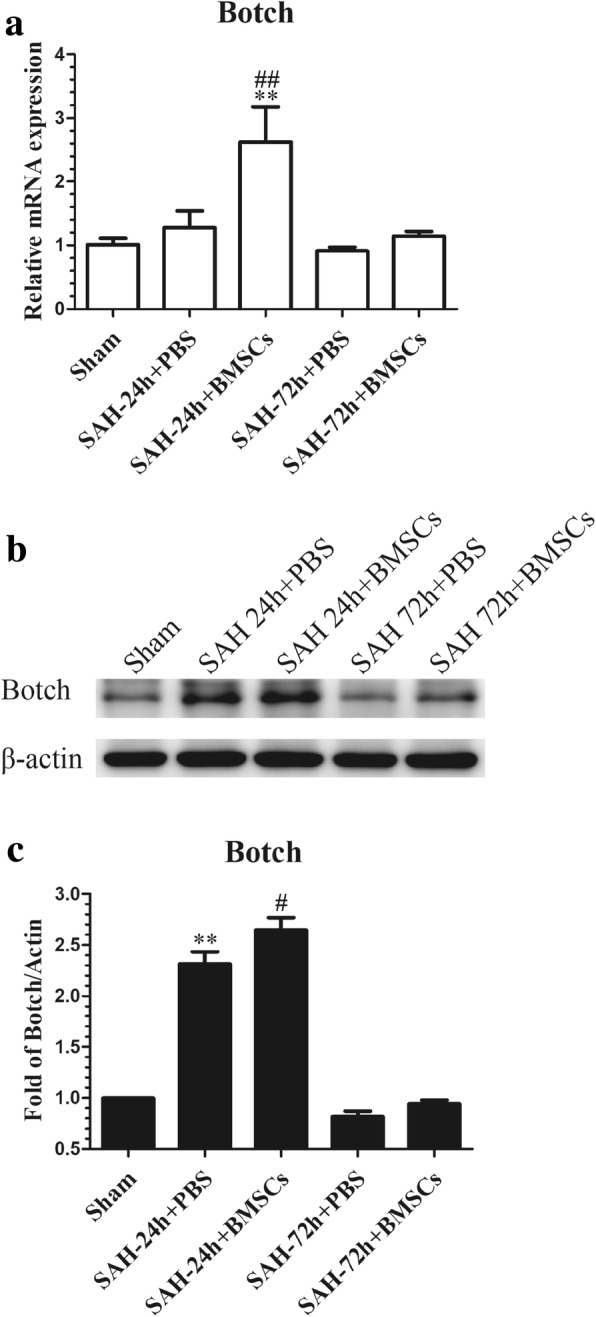
Effects of BMSCs treatment on the expression of Botch in brain tissue at 24 and 72 h post-SAH. The qRT-PCR analysis of the Botch mRNA level showed that Botch was upregulated in SAH + BMSCs group compared with SAH + PBS group at 24 h post-SAH (**a**); *n* = 3 in each group; data expressed as the mean ± SEM. **P < 0.01 versus Sham, ^##^*P* < 0.01 versus SAH + PBS group. Western blot data showed that BMSCs treatment significantly increased the protein levels of Botch in left cortex at 24 h post-SAH (**b**); *n* = 6 in each group; data are expressed as the mean ± SEM. **P < 0.01 versus Sham, ^#^*P* < 0.05 versus SAH + PBS group (**c**). The qRT-PCR and Western blotting analysis of Botch protein and mRNA levels in the left cortex showed no obvious upregulation in BMSCs treatment group at 72 h post-SAH

It has been reported that Botch can inhibit Notch signaling both in vitro and in vivo [47]. To confirm whether Botch participated in the protective effects of BMSCs on EBI after SAH, we examined the expression of Botch after SAH with and without BMSCs treatment. Results showed that protein and mRNA levels of Botch were upregulated at 24 h post-SAH compared with the sham group but not at 72 h post-SAH. BMSCs treatment significantly increased mRNA and protein levels of Botch compared with the SAH group (Fig. 10a–c).

### Botch knockdown in BMSCs fully abolished the protective effects of BMSCs at 24 h post-SAH

**Fig. 11:**
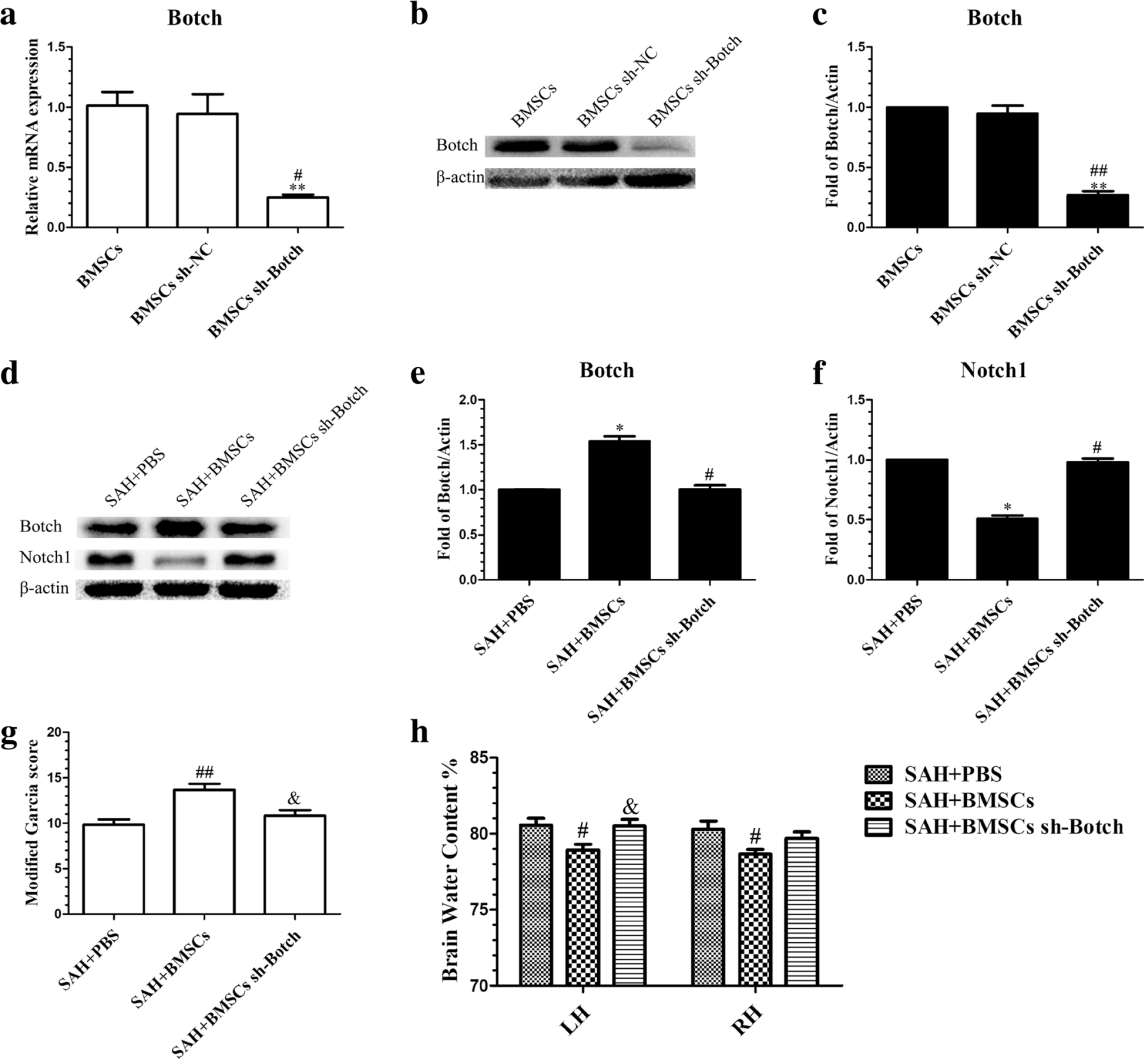
Botch shRNA-mediated knockdown of Botch in BMSCs abolished their protection of EBI after SAH. The efficiency of shRNA-mediated knockdown of Botch in BMSCs was confirmed by qRT-PCR (**a**) (*n* = 3/group) and Western blotting (**b**) (*n* = 6/group). Data are expressed as the mean ± SEM. **P < 0.01 versus BMSCs group, ^#^*P* < 0.05, ^##^*P* < 0.01versus BMSCs sh-NC group (**c**). Representative images of Western blot data showing the expression of Botch and Notch1 in SAH + PBS, SAH + BMSCs, and SAH + BMSCs sh-Botch groups (**d**). Quantitative analyses of Botch (**e**) and Notch1 (**f**) expression indicating that BMSCs sh-Botch transplantation significantly decreased Botch expression in brain tissue at 24 h post-SAH but increased Notch1 expression; *n* = 6 in each group; data are expressed as the mean ± SEM. *P < 0.05 versus SAH + PBS group, ^#^*P* < 0.05 versus SAH + BMSCs group. BMSCs sh-Botch treatment accelerated neurobehavioral deficits (**g**) and BWC (**h**) compared with BMSCs treatment group; *n* = 6 in each group; data are expressed as the mean ± SEM. ^#^*P* < 0.05, ^##^*P* < 0.01 versus SAH + PBS group, ^&^*P* < 0.05 versus SAH + BMSCs group

To determine if Botch is required for EBI protection by BMSCs after SAH, we knocked down Botch expression in BMSCs. The qRT-PCR and Western blotting were performed to confirm that Botch expression in BMSCs was attenuated after knockdown with Botch shRNA (Fig. 11a–c). Botch knockdown BMSCs treatment abolished the effects of BMSCs on upregulation of Botch and inhibition of Notch1 levels at 24 h post-SAH (Fig. 11d–f). The attenuation of neurobehavioral deficits (Fig. 11g) and reduction in BWC (Fig. 11h) associated with BMSCs treatment was also negated following knockdown of Botch in BMSCs.

## Discussion

In this study, we revealed that the beneficial effects of BMSCs on EBI and neuroinflammation after SAH may be attributed to inhibition of Notch1-mediated microglial activation and neuronal degeneration by Botch. Our experiments demonstrated four important findings as follows: (1) BMSCs treatment improved neurobehavioral deficits, decreased the BWC, and ameliorated BBB disruption in EBI after SAH; (2) endogenous Notch1 expression was upregulated in a time-dependent manner and peaked at 24 h post-SAH. Inhibition of the Notch1 signaling pathway by DAPT or Notch1 siRNA suppressed activated microglia-mediated neuroinflammation and inhibited the expression of inflammatory factors, thereby ameliorating EBI after SAH; (3) BMSCs attenuated the inflammatory response at least partially due to inhibition of Notch1 signaling, which subsequently repressed NF-κB phosphorylation at 24 and 72 h post-SAH; and (4) shRNA-mediated knockdown of Botch in BMSCs reversed BMSCs-induced inhibition of Notch1 signaling and abolished the beneficial effects of BMSCs on EBI after SAH.

A growing body of clinical and preclinical studies suggests that the inflammatory response occurs early after SAH and contributes to the progression of SAH-induced EBI [61–63]. As the resident immunocompetent and phagocytic cells of the CNS, activated microglia, which produce inflammatory mediators, play a significant role in neuroinflammation [10, 60]. Thus, inhibition of microglial activation is beneficial to prevent deleterious inflammation and to alleviate inflammation-induced EBI after SAH [52].

In recent years, the beneficial effects of MSCs transplantation on various diseases had been attributed to parenchymal cell replacement, trophic factor secretion, and regulation of immunity [64–67]. BMSCs have been reported to ameliorate inflammation-induced brain injury in different CNS disease [14, 67–69] by suppressing microglial activation and inhibiting proinflammatory cytokines production. Consistent with these previous studies, our experimental results showed that BMSCs transplantation ameliorated brain edema, neurobehavioral deficits, and neuronal injury, as well as inhibited microglial activation; decreased the expression of proinflammatory factors including IL-6, IL-1β, and TNF-α; and increased the expression of the anti-inflammatory factor, IL-10, in EBI after SAH. Nevertheless, the underlying molecular mechanisms of the anti-inflammatory and neuroprotective functions of BMSCs on EBI needed further investigation.

Previous reports showed that Notch1 signaling exerted a critical role in the differentiation, proliferation, and apoptosis of cells during development and in adult tissues [28, 32]. On binding to ligands, Notch1 is cleaved by furin-like convertase at the extracellular domain after which the transmembrane domain is incised by metalloproteases and the γ-secretase enzyme complex [70]; the NICD is translocated to the nucleus, where it regulates transcription of Notch1 target genes. Recent studies demonstrated that the Notch1 signaling pathway has an essential role in the regulation of the inflammatory response in different pathological settings [34, 35, 71, 72]. In the CNS, Notch1 signaling not only regulates microglia-mediated neuroinflammation [36–38] but also participates in the process of neuronal injury in ischemic stroke [40]. Our study revealed that Notch1 expression was significantly increased and peaked at 24 h post-SAH and that the NICD was primarily localized in microglia. Based on these results, we speculated that Notch1 signaling pathway activation possibly enhanced microglial activation, thus upregulating proinflammatory cytokines, which may have exacerbated EBI after SAH. DAPT, as a γ-secretase inhibitor, can suppress Notch1 signaling activation, with several studies demonstrating that DAPT ameliorated brain damage and neurological deficits and inhibited proinflammatory cytokine secretion and microglial activation in an ischemic stroke model [39, 41]. Consistent with the above findings, our results showed that DAPT administration significantly inhibited the expression of Notch1 and its downstream factors, including Notch1, NICD, RBP-Jκ, and Hes-1 compared with SAH + DMSO group after SAH. DAPT treatment also suppressed the microglia-mediated inflammatory reaction, alleviated brain edema and neuronal injury, and improved neurobiological deficits at 24 h post-SAH.

Recent studies reported that Notch1 enhanced the inflammatory reaction [35, 41] and aggravated the neuronal degeneration [40], at least in part, by augmenting NF-κB phosphorylation; our results indicated that the increased expression of phosphorylated NF-κB after SAH was suppressed after DAPT treatment. In addition, we used Notch1 siRNA to confirm the functions of Notch1 signaling in the microglia-mediated inflammatory response in EBI after SAH. Our data showed that Notch1 siRNA successfully knocked down the expression of Notch1 signaling and decreased microglial activation and neuronal degeneration; as well as ameliorated cerebral edema and neurobehavioral deficits. Protein levels of p-NF-κB were also reduced after Notch1 knockdown 48 h prior to SAH. Based on these results, we speculate that Notch1 signaling may participate in the neuroinflammatory reaction and brain injury in EBI after SAH by regulating the phosphorylation of NF-κB.

It has been reported that BMSCs transplantation enhanced neurogenesis after ischemic stroke [42] and improved osteopenia in Lupus [44] via inhibiting Notch1 signaling. We therefore speculated that BMSCs treatment alleviated microglia-mediated neuroinflammation and neuronal injury in EBI after SAH partly by inhibition of the Notch1 signaling pathway. In the present study, our findings showed that Notch1 signaling was significantly suppressed by BMSCs at 24 h and 72 h post-SAH, which was confirmed by measuring protein and mRNA levels of Notch1, NICD, RBP-Jκ, and Hes-1. Double immunofluorescence staining also revealed that the expression of NICD in activated microglia was decreased after BMSCs treatment. Additionally the expression of p-NF-κB was significantly inhibited after BMSCs administration. Taken together, these results indicated that BMSCs may ameliorate neuroinflammation in EBI after SAH through inhibition of Notch1 signaling and NF-κB phosphorylation.

Previous study demonstrated that overexpression of Botch in vivo alleviated neuronal apoptosis and inflammation in intracerebral hemorrhage [48]. We hypothesized that BMSCs transplantation inhibited Notch1 signaling by increasing Botch expression in brain tissue; we first detected Botch protein and mRNA levels in BMSCs, with results indicating that Botch strongly expressed in cultured BMSCs. Then we detected levels of Botch expression after SAH with and without BMSCs treatment, and showed that Botch expression was slightly increased compared with the sham group and that BMSCs treatment significantly increased the expression of Botch in the ipsilateral hemisphere. Finally, we knocked down Botch in BMSCs and administrated these cells intravenously after SAH. The results indicated that knockdown of Botch in BMSCs abrogated beneficial effects of BMSCs on EBI after SAH and that the inhibition of Notch1 signaling was also abolished. These results suggested that the neuroprotective and anti-inflammatory effects of BMSCs were partly attributed to the upregulation of Botch expression which resulted in suppression of the Notch1 signaling pathway.

There are several points in our study that need to be discussed. First, although EBI as a new pathological mechanism has been explored in recent years, cerebral vasospasm remains a cause of morbidity and mortality after aneurismal SAH. Previous studies suggested that BMSCs transplantation exerted other protective functions, such as promote angiogenesis, prevent endothelial damage, and enlarge the diameters of the basilar artery and pial vessels via differentiating into endothelial cells and secreting functional molecules in ischemic stroke [72–74]; our study only investigated the anti-inflammatory effects of BMSCs on EBI after SAH, therefore we cannot rule out the possible vascular effects of BMSCs on cerebral vasospasm-mediated ischemic injury after SAH. Second, only an immediate post-SAH intravenous injection of BMSCs was tested; the effects of multiple systemic BMSCs treatment at different time course on long-term outcome should be examined. Finally, further experiments of BMSCs treatment combined with Notch1 agonists and inhibitors should be performed to better demonstrate that BMSC treatment alleviated neurobehavioral impairments and the inflammatory response in EBI after SAH via upregulation of Botch to suppress Notch1 signaling.

## Conclusions

This study demonstrated that the neuroprotective effects of BMSCs in the treatment of EBI after SAH were partly due to Botch upregulation in brain tissue, which inhibited Notch1 signaling-induced NF-κB phosphorylation and then alleviated microglia-mediated neuroinflammation (Additional file 1). Therefore, BMSCs transplantation may provide a promising therapeutic method against EBI after SAH.

## Additional files

Additional file 1: The experimental design and schematic diagram. Six experiments were conducted in our study. The schematic diagram showed the potential mechanism of BMSCs mediated anti-inflammatory effects on EBI after SAH. Briefly, Notch1 receptors are expressed on microglial cells. Following SAH ictus, Notch1 is activated by proteolytic cleavage, thus releasing NICD into the cytoplasm, which in turn enhances NF-κB activation. In addition, NICD can translocate to the nucleus and combine with RBP-Jκ to generate co-activator complex to promote the transcription of NF-κB and augment the inflammatory response. Botch efficiently antagonized the proteolytic cleavage and activation of the Notch1 receptor. BMSCs treatment upregulated Botch in brain tissue and alleviated the Notch1-driven microglia-mediated inflammatory response. (PDF 88 kb)

Additional file 2: Co-staining of NeuN and CD68 as well as NueN and Iba1 at 24 h post-SAH. Representative images of double immunofluorescence staining for NeuN and CD68 or Iba1, results showed that Iba1- and CD68-positive cells were increased, but the numbers of NeuN-positive neurons were decreased in the cortex of left hemisphere after 24 h SAH. (TIF 8397 kb)

Additional file 3: Effects of DAPT, Notch1 siRNA and BMSCs on (Nuclear factor-κB) NF-κB phosphorylation after SAH. DAPT administration inhibited p-NF-κB expression at 24 h post-SAH. Representative western blot bands (A) and quantification of p-NF-κB in the left cortex (B) indicate that DAPT inhibited NF-κB phosphorylation at 24 h post-SAH; *n* = 6 in each group; data are expressed as the mean ± SEM. ^*^P < 0.05 versus Sham, ^###^*P* < 0.001 versus SAH + DMSO group. Notch1 siRNA injection inhibited phosphorylation of NF-κB at 24 h post-SAH. Representative western blot bands (C) and quantitative analyses of p-NF-κB expression in the left cortex (D) show that knockdown Notch1 inhibited NF-κB phosphorylation at 24 h post-SAH; n = 6 in each group; data are expressed as the mean ± SEM. ^*^P < 0.05 versus Sham, ^###^*P* < 0.001 versus SAH + Scramble siRNA group. BMSCs treatment suppressed NF-κB phosphorylation at 24 and 72 h post-SAH. Western blot data showed that BMSCs significantly suppressed the phosphorylation of NF-κB (E and F) at 24 and 72 h post-SAH; n = 6 in each group; data are expressed as the mean ± SEM. ^*^P < 0.05 versus Sham, ^#^*P* < 0.05 versus SAH + PBS group. (TIF 4601 kb)

## Abbreviations

ANOVA: Analysis of variance
BBB: Blood-brain barrier
BMSCs: Bone marrow mesenchymal stem cells
BWC: Brain water content
CD: Cluster of differentiation
Chac1: Cation transport regulator homolog 1
CNS: Central nervous system
DAPT: *N*-[*N*-(3,5-difluorophenacetyl-l-alanyl)]-S-phenylglycine *t*-butyl ester
DMSO: Dimethylsulfoxide
DW: Dry weight
EBI: Early brain injury
ECA: External carotid artery
ELISA: Enzyme-linked immunosorbent assay
FJC: Fluoro-Jade C
H&E: Hematoxylin and eosin
Hes-1: Hes family basic helix loop helix transcription factor 1
Iba1: Ionized calcium binding adapter molecule1
ICA: Internal carotid artery
IF: Immunofluorescence
IHC: Immunohistochemistry
IL: Interleukin
MRI: Magnetic resonance imaging
MSCs: Mesenchymal stem cells
NC: Negative control
NF-κB: Nuclear factor-κB
NICD: Notch1 intracellular receptor domain
NPG7: Neuroprotective gene 7
PBS: Phosphate buffer solution
P-NF-κB: Phosphorylation of nuclear factor-κB
qRT-PCR: Quantitative real-time polymerase chain reaction
RBP-Jκ: Recombining binding protein suppressor of hairless
RT: Room temperature
SAH: Subarachnoid hemorrhage
SEM: Standard error of the mean
shRNA: Short hairpin RNA
siRNA: Small interfering RNA
TE: Echo time
TNF: Tumor necrosis factor
TR: Repetition time
WW: Wet weight
