# Supplementary figures and images for: Mesenchymal stem cells alleviate the early brain injury of subarachnoid hemorrhage partly by suppression of Notch1-dependent neuroinflammation: involvement of Botch
Source: J Neuroinflammation. 2019 Jan 15;16:8. doi: 10.1186/s12974-019-1396-5 (PMC6334441; doi:10.1186/s12974-019-1396-5)

## Experiment 1

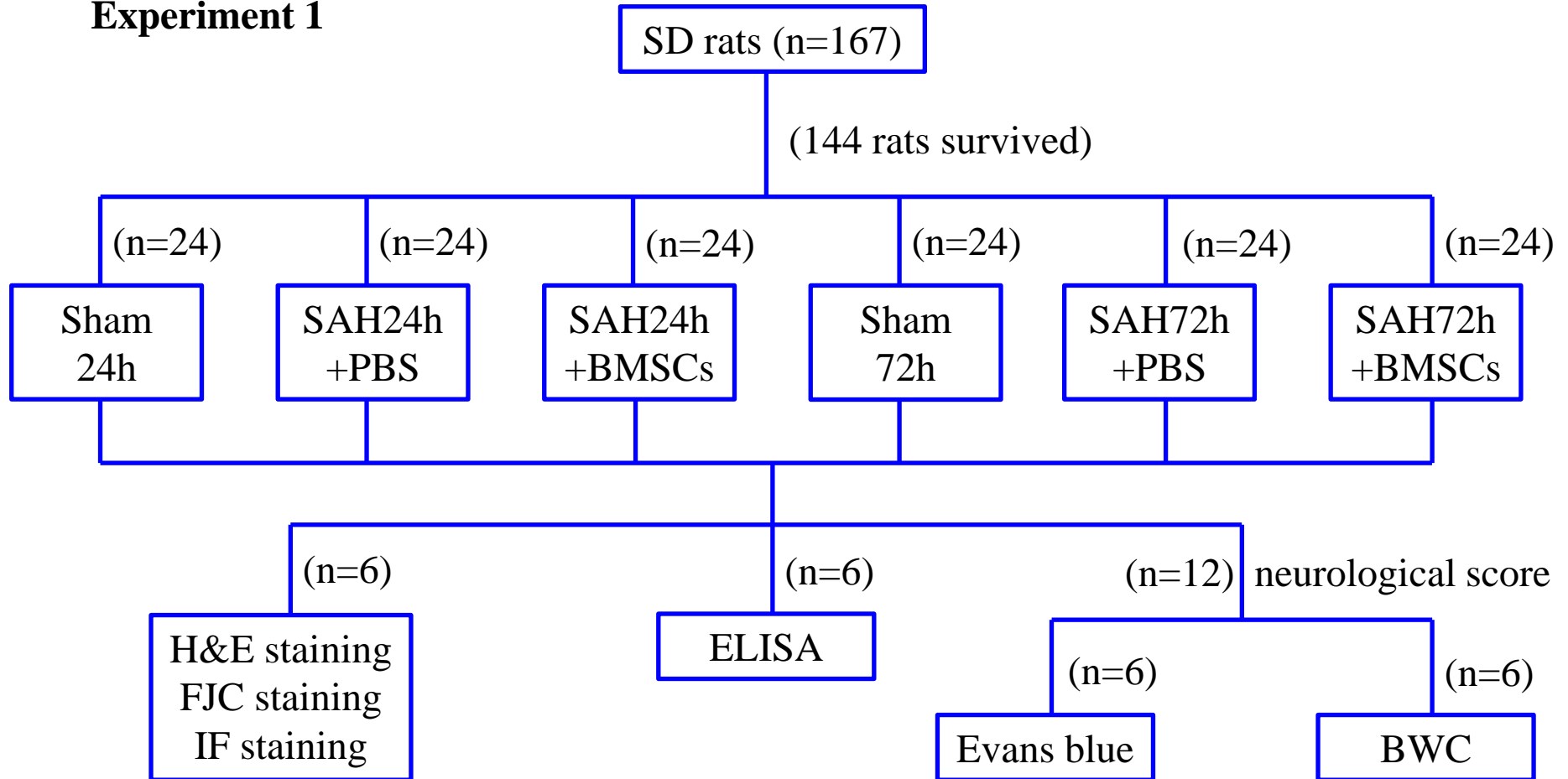

## Experiment 2

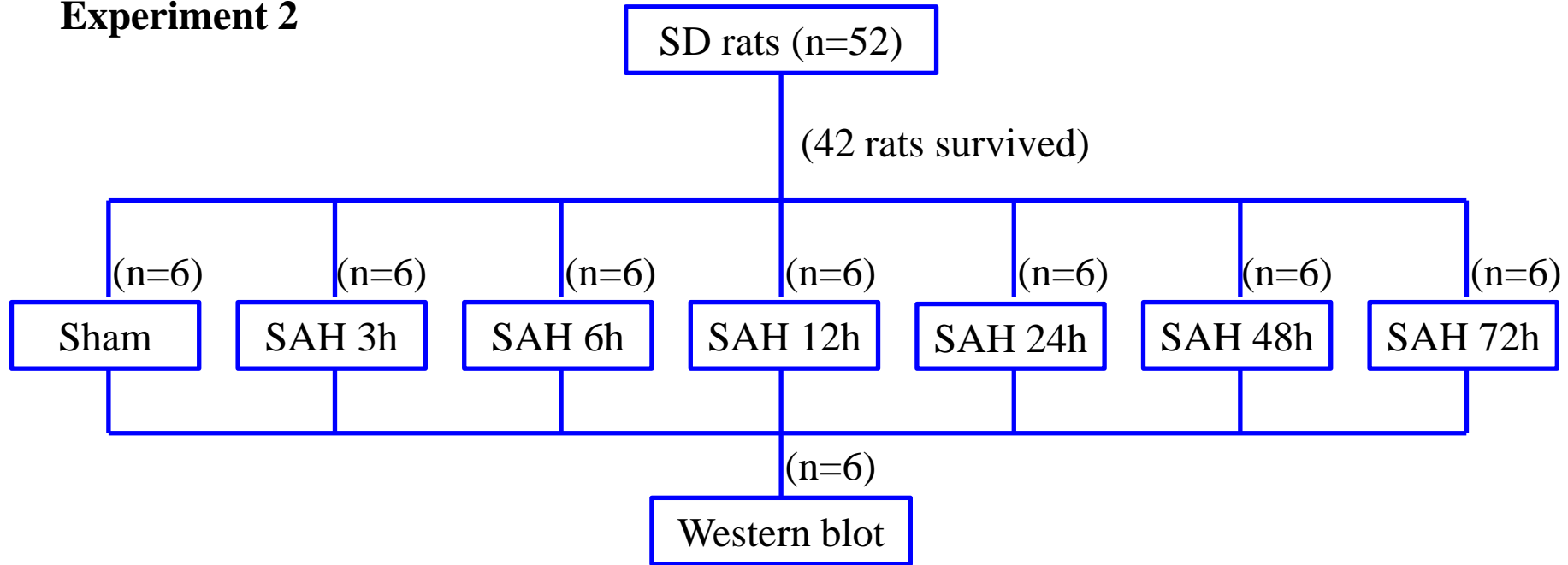

## Experiment 3

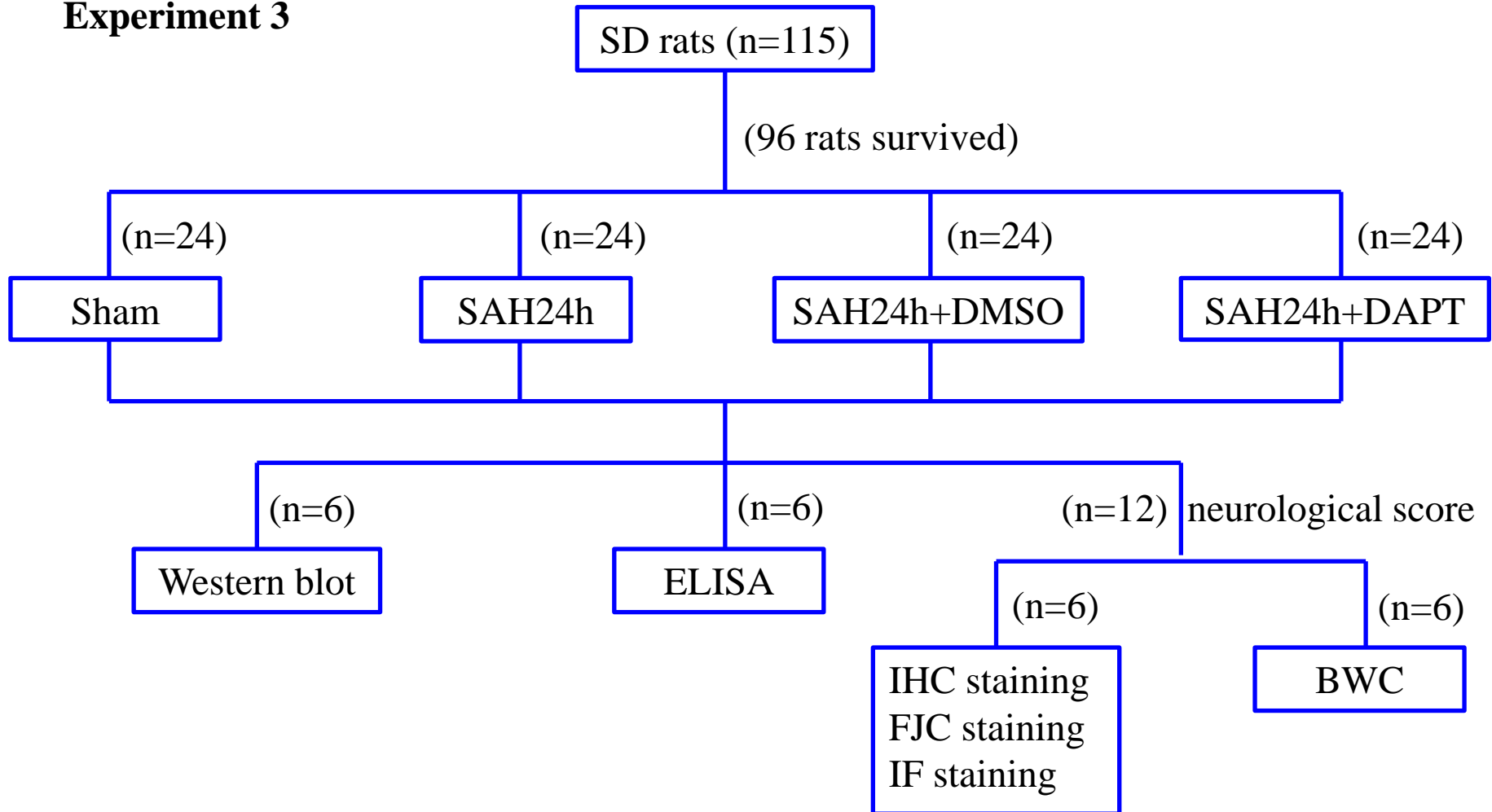

## Experiment 4

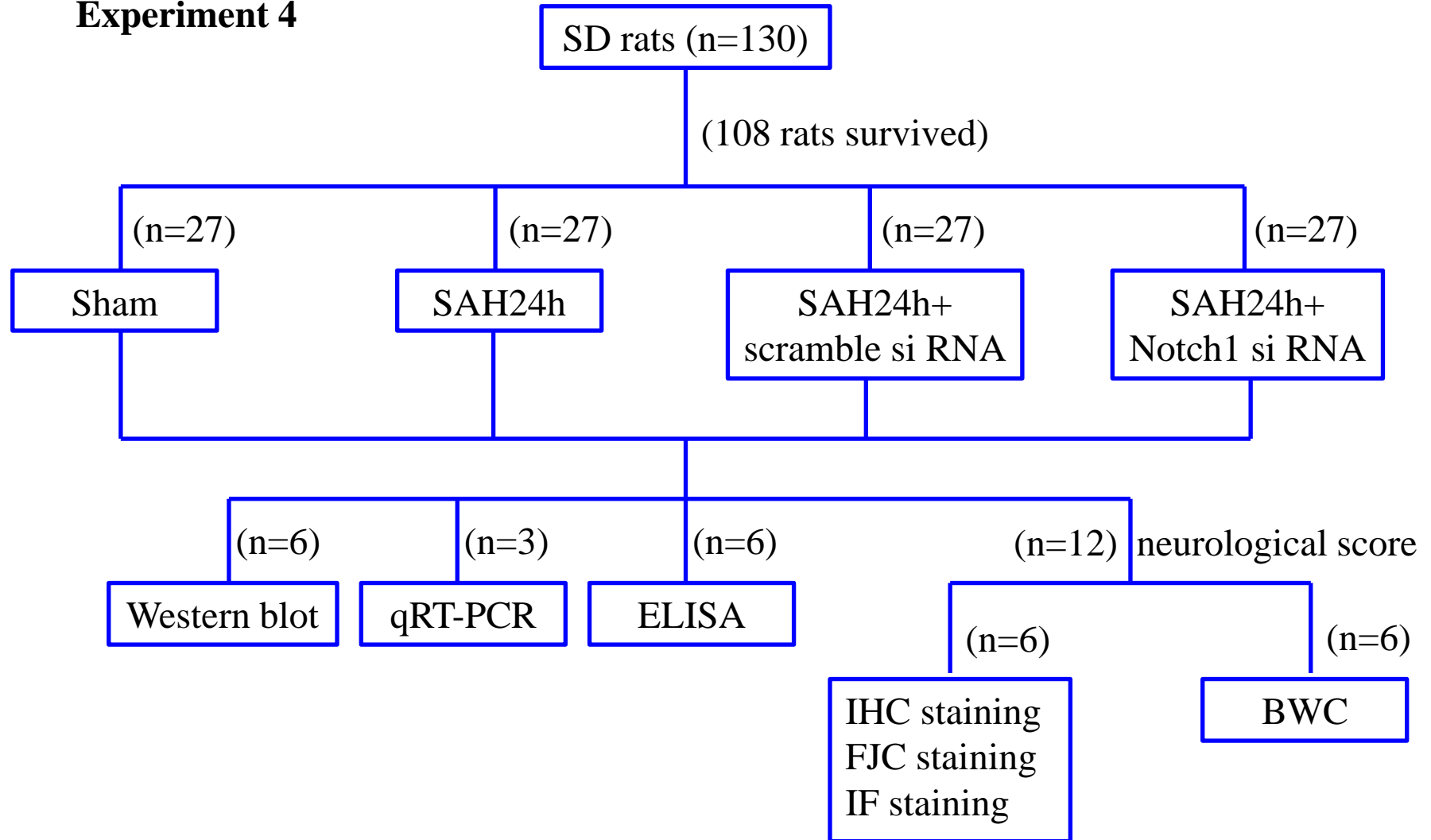

## Experiment 5

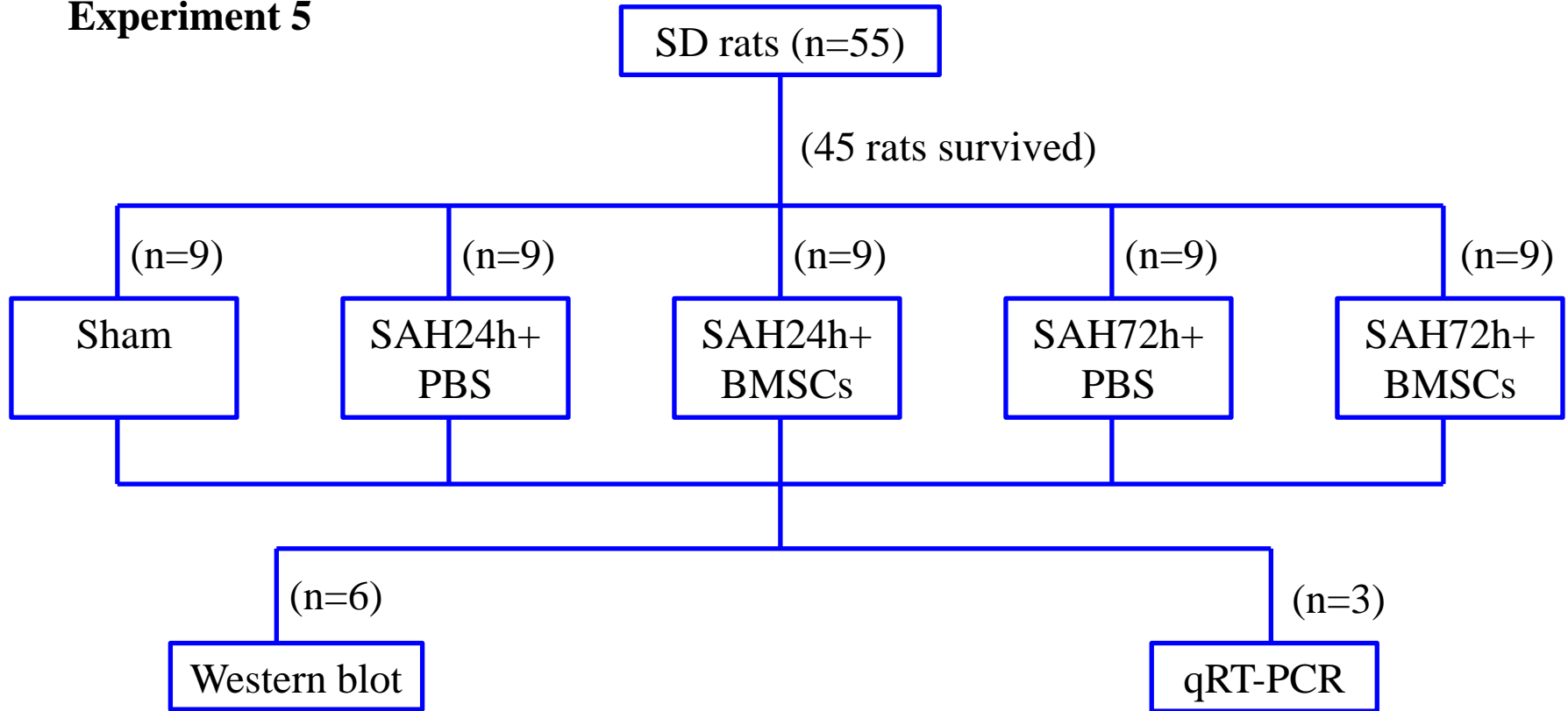

## Experiment 6

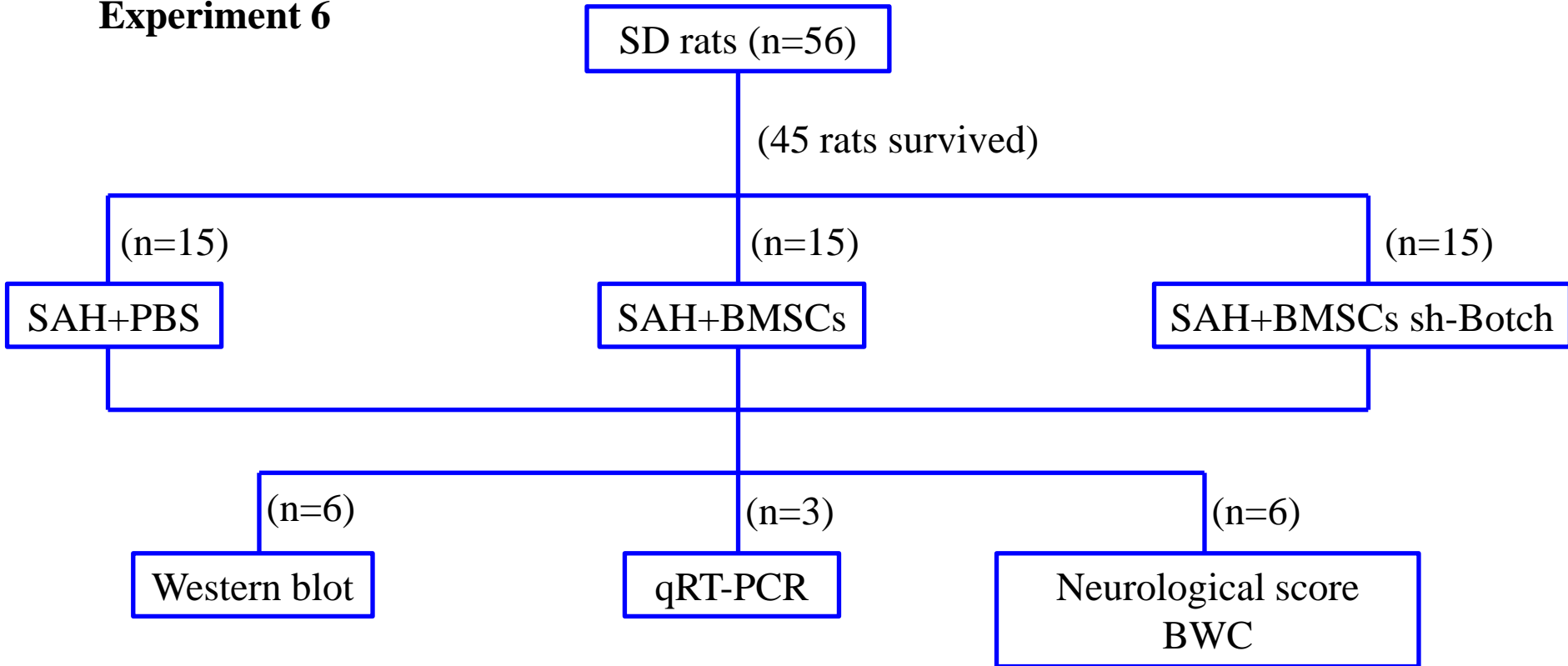

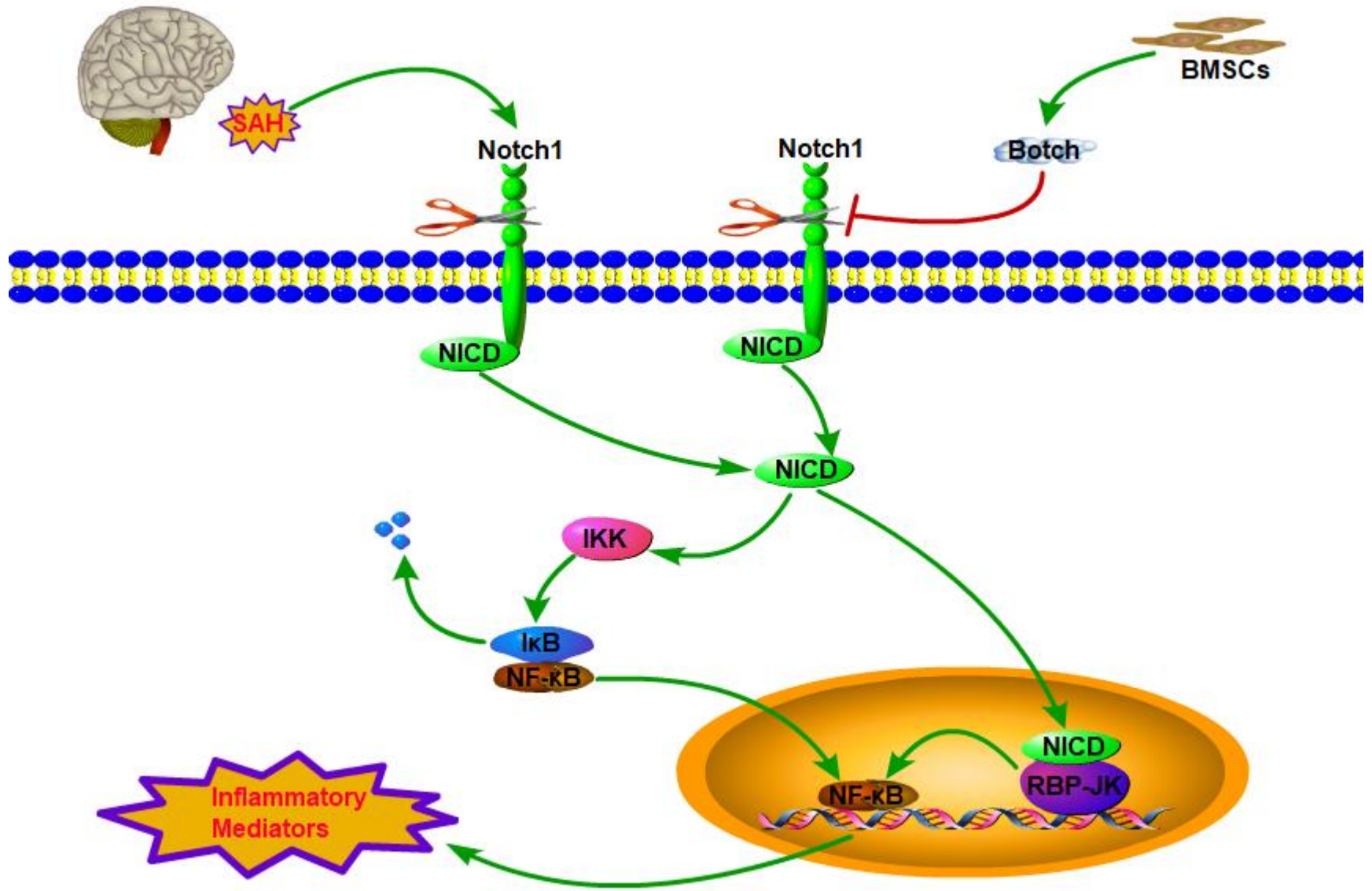

Supplement: Supplementary file 1 — The experimental design and schematic diagram. Six experiments were conducted in our study. The schematic diagram showed the potential mechanism of BMSCs mediated anti-inflammatory effects on EBI after SAH. Briefly, Notch1 receptors are expressed on microglial cells. Following SAH ictus, Notch1 is activated by proteolytic cleavage, thus releasing NICD into the cytoplasm, which in turn enhances NF-κB activation. In addition, NICD can translocate to the nucleus and combine with RBP-Jκ to generate co-activator complex to promote the transcription of NF-κB and augment the inflammatory response. Botch efficiently antagonized the proteolytic cleavage and activation of the Notch1 receptor. BMSCs treatment upregulated Botch in brain tissue and alleviated the Notch1-driven microglia-mediated inflammatory response. (PDF 88 kb) [file 12974_2019_1396_MOESM1_ESM.pdf]

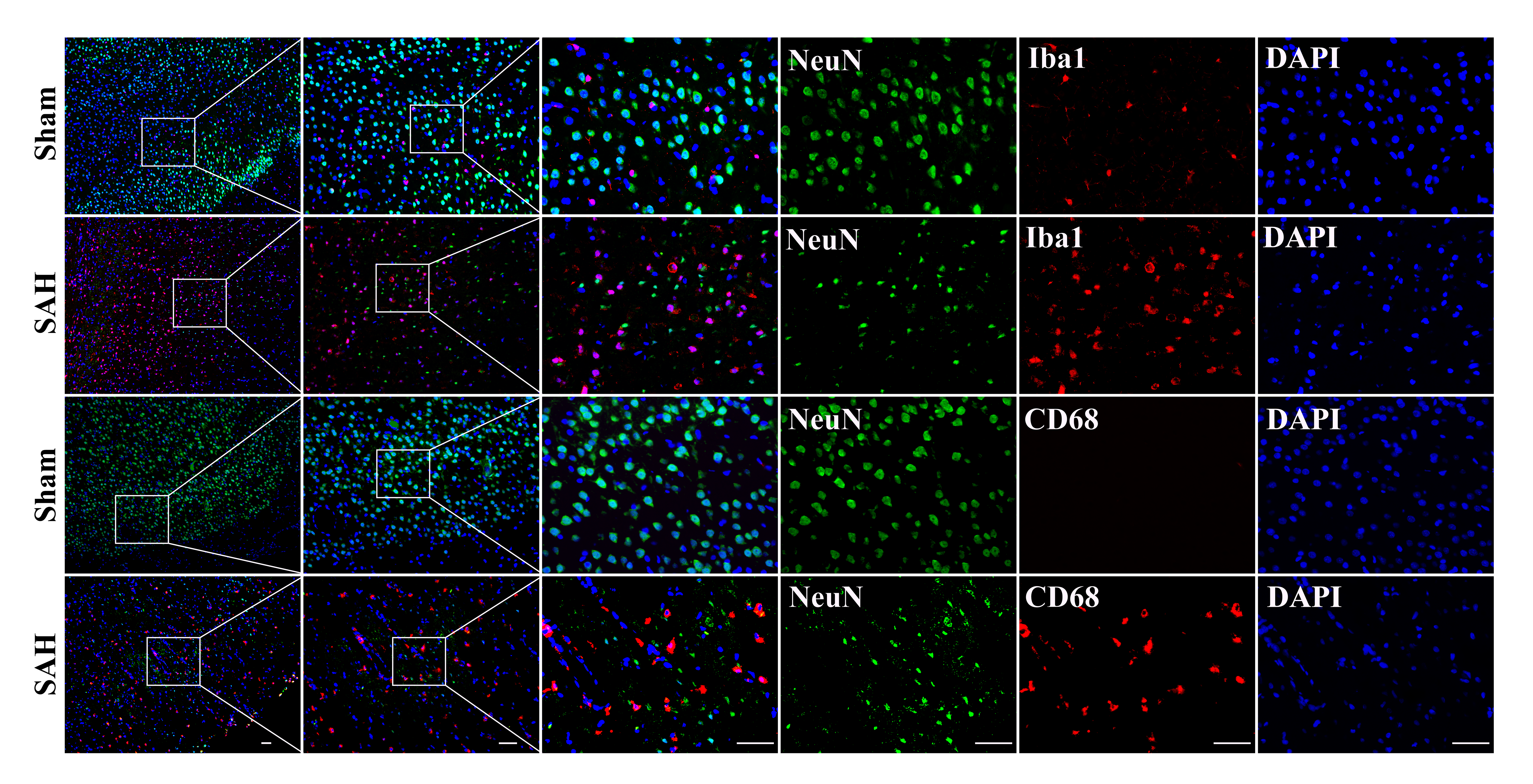

Supplement: Supplementary file 2 — Co-staining of NeuN and CD68 as well as NueN and Iba1 at 24 h post-SAH. Representative images of double immunofluorescence staining for NeuN and CD68 or Iba1, results showed that Iba1- and CD68-positive cells were increased, but the numbers of NeuN-positive neurons were decreased in the cortex of left hemisphere after 24 h SAH. (TIF 8397 kb) [file 12974_2019_1396_MOESM2_ESM.tif]
